# Biomarker-Driven Approaches to Bone Metastases: From Molecular Mechanisms to Clinical Applications

**DOI:** 10.3390/biomedicines13051160

**Published:** 2025-05-10

**Authors:** Youssef Elshimy, Abdul Rahman Alkhatib, Bilal Atassi, Khalid S. Mohammad

**Affiliations:** Department of Anatomy, College of Medicine, Alfaisal University, Riyadh 11533, Saudi Arabia; yelshimy@alfaisal.edu (Y.E.); aralkhatib@alfaisal.edu (A.R.A.); batassi@alfaisal.edu (B.A.)

**Keywords:** bone metastasis, biomarkers in precision oncology, circulating biomarkers, CTCs, ctDNA, BTMs, therapeutic targets, precision medicine

## Abstract

Bone metastases represent a critical complication in oncology, frequently indicating advanced malignancy and substantially reducing patient quality of life. This review provides a comprehensive analysis of the complex interactions between tumor cells and the bone microenvironment, emphasizing the relevance of the “seed and soil” hypothesis, the RANK/RANKL/OPG signaling axis, and Wnt signaling pathways that collectively drive metastatic progression. The molecular and cellular mechanisms underlying the formation of osteolytic and osteoblastic lesions are examined in detail, with a particular focus on their implications for bone metastases associated with breast, prostate, lung, and other cancers. A central component of this review is the categorization of pathological biomarkers into four types: diagnostic, prognostic, predictive, and monitoring. We provide a comprehensive evaluation of circulating tumor cells (CTCs), bone turnover markers (such as TRACP-5b and CTX), advanced imaging biomarkers (including PET/CT and MRI), and novel genomic signatures. These biomarkers offer valuable insights for early detection, enhanced risk stratification, and optimized therapeutic decision-making. Furthermore, emerging strategies in immunotherapy and bone-targeted treatments are discussed, highlighting the potential of biomarker-guided precision medicine to enhance personalized patient care. The distinctiveness of this review lies in its integrative approach, combining fundamental pathophysiological insights with the latest developments in biomarker discovery and therapeutic innovation. By synthesizing evidence across various cancer types and biomarker categories, we provide a cohesive framework aimed at advancing both the scientific understanding and clinical management of bone metastases.

## 1. Introduction

### 1.1. Overview of Bone Metastases

Bone metastases constitute a significant clinical challenge in cancer management, exhibiting a complex epidemiological landscape and profound clinical implications. As cancer spreads, the bone becomes a frequent site for metastasis, particularly in malignancies such as breast, prostate, and lung cancer. It has been reported that approximately 50% of patients with advanced breast cancer will develop clinically detectable osteolytic metastases, while the incidence increases to over 75% in autopsy studies [1]. Additionally, bone metastases are associated with various solid tumors, where the incidence rates can vary; certain cancers demonstrate a predilection for bone involvement. For instance, a study in the United States identified a clear association between solid tumors and the occurrence of bone metastases, highlighting their role as a significant prognostic indicator linked to shorter survival rates following detection [2].

The clinical relevance of bone metastases extends beyond mere presence; they have a significant impact on patient quality of life and survival outcomes. Evidence suggests that patients with bone metastases from solid tumors experience shorter overall survival compared to those without [2,3]. The underlying mechanisms often involve complex interactions between tumor cells and the bone microenvironment, leading to bone destruction and subsequent skeletal-related events (SREs) such as fractures and pain [4,5]. This is particularly evident in breast cancer, where tumor cells activate osteoclast-mediated bone resorption, exacerbating skeletal fragility [6]. Additionally, specific biological markers, such as alkaline phosphatase, serve as prognostic factors in assessing disease progression and treatment response in bone metastases [7].

The prognosis associated with bone metastases varies by cancer type and metastatic site. For instance, patients with bone metastases from intrahepatic cholangiocarcinoma had an incidence rate of 14.5%, with single-site metastases associated with relatively better outcomes compared to those with widespread disease [8]. Furthermore, the distinct biology of prostate cancer-associated bone metastases, characterized by a propensity for osteoblastic activity, poses unique treatment challenges and necessitates tailored therapeutic strategies [9]. Recent advances in targeted therapies, including inhibitors of the insulin-like growth factor (IGF) pathway, show promise in managing bone metastases and reducing associated morbidity [10].

The evolving understanding of bone metastatic disease underscores the need for ongoing research and enhanced clinical protocols to alleviate the substantial burdens that patients face. Comprehensive epidemiological studies and advancements in biomarker discovery are crucial for enhancing early detection and tailoring treatment approaches to individual patient profiles [11].

### 1.2. Precision Medicine Paradigm and Role of Biomarkers in Tailoring Individualized Treatment

The Precision Medicine Paradigm represents a significant transformation in the field of oncology, emphasizing the customization of treatment approaches to individual patients based on their distinct biological and genomic characteristics. This transformation is made possible by advancements in molecular biology, which enable clinicians to categorize cancer with unparalleled precision. The essential goal of precision medicine in cancer therapy is to amplify treatment efficacy while simultaneously alleviating adverse effects, thereby fostering improved patient outcomes and reducing overall healthcare costs [12,13]. Clinicians can make more informed treatment decisions by utilizing biomarkers—specific indicators found within the patient’s biology. These biomarkers can include genetic mutations, protein expressions, and epigenetic modifications, all of which can influence the effectiveness of cancer therapies [13,14].

Precision medicine holds significant importance in oncology, as evidenced by its contribution to the development of targeted therapies that operate on specific molecular pathways associated with cancer development. For instance, solving the genetic structure of tumors leads to the identification of viable targets, which in turn supports the rollout of more personalized and successful treatment methods [15,16]. Progressions in genomic sequencing technologies have made identifying these targets feasible, resulting in notable enhancements in managing diverse cancer types, including non-small-cell lung cancer and urothelial cancer, where customized treatment strategies have become the norm [16,17].

Biomarkers play a critical role in the precision medicine framework. They not only serve as targets for therapy but also guide the use of companion diagnostics, which are tests designed to determine the suitability of particular treatments for individual patients [15]. Furthermore, the presence of specific biomarkers can help predict responses to therapies, thereby avoiding ineffective treatments and minimizing adverse effects, which is especially valuable given the heterogeneity of cancer responses among patients [18]. For example, in the case of advanced non-small-cell lung cancer, clinical practice gaps in biomarker testing have been identified, emphasizing the need for better integration of genomic information into treatment plans to fully realize the potential of precision medicine [17].

## 2. Pathophysiology of Bone Metastases

### 2.1. Bone Microenvironment and Tumor Interactions

**Figure 1:**
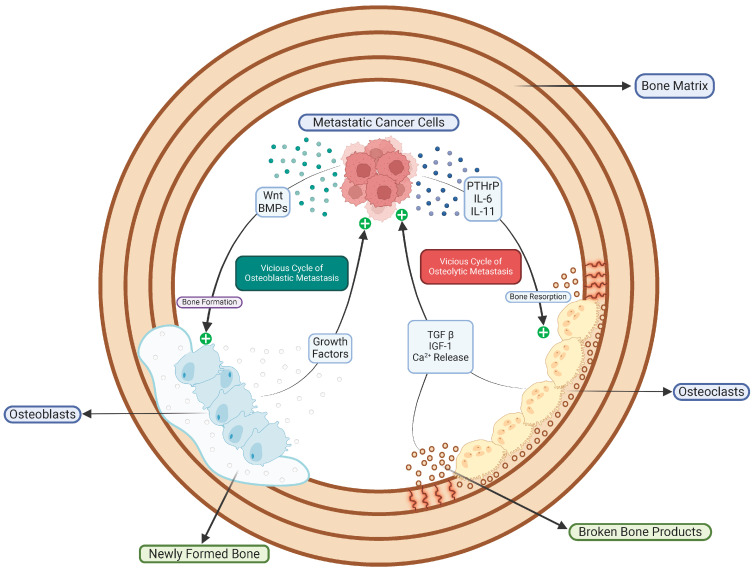
Schematic representation of the vicious cycle of bone metastasis. This figure illustrates the dynamic interaction between metastatic cancer cells and the bone microenvironment, emphasizing the dual nature of bone remodeling in metastasis. Metastatic cancer cells secrete various factors, including parathyroid hormone-related protein (PTHrP), interleukin-6 (IL-6), and interleukin-11 (IL-11), which stimulate osteoclasts to enhance bone resorption. This resorption releases growth factors such as transforming growth factor-beta (TGF-β), insulin-like growth factor 1 (IGF-1), and calcium, further promoting tumor growth and metastatic activity. Simultaneously, tumor-secreted factors such as Wnt proteins and bone morphogenetic proteins (BMPs) stimulate osteoblasts, leading to new bone formation, but structurally flawed and disorganized, characteristic of osteoblastic metastasis. The figure encapsulates the interconnected cycles of osteolytic and osteoblastic metastasis that contribute to the progression of bone metastases and highlights potential therapeutic targets within these pathways.

The pathophysiology of bone metastasis is central to the “seed and soil” hypothesis, which suggests that circulating tumor cells (the seeds) preferentially target the bone microenvironment (the soil) [19,20]. This concept highlights the intricate networks that form between spreading cancers and the distinct structure of the bone marrow, which facilitates metastatic colonization (Figure 1). The bone matrix, rich in growth factors and featuring a specialized vascular system, along with its ability to remodel, creates a favorable setting for tumor cell attachment and expansion [20,21]. Recent research indicates that tumor-derived extracellular vesicles prepare the premetastatic niche by influencing the differentiation of stromal cells and attracting immune cells, enhancing the bone microenvironment’s susceptibility to metastatic invasion [19,22].

Bone metastases are classified based on radiological findings into either osteolytic or osteoblastic lesions [23]. Osteolytic lesions arise mainly from osteoclast-mediated bone resorption. Tumor cells secrete parathyroid hormone-related protein (PTHrP), interleukin-6 (IL-6), and interleukin-11 (IL-11), which stimulate osteoclast differentiation and activity [22,24]. This pathological process establishes a damaging cycle; the breakdown of the bone matrix releases TGF-β and additional mitogens, further promoting tumor growth and enhancing osteoclast activation [20,24]. Conversely, the osteoblastic lesions seen in prostate cancer metastases result from tumor-driven changes in bone formation pathways. Here, cancer cells release Wnt family members and bone morphogenetic proteins (BMPs), leading to increased osteoblast differentiation and the formation of sclerotic bone [20,21]. Additionally, tumor–stromal interactions within the bone microenvironment complicate the situation by allowing osteolytic and osteoblastic activities to coexist in mixed metastases [20].

Bone marrow stromal cells, particularly mesenchymal stem cells (MSCs), play a crucial role in metastasis progression. They do so through direct interactions and paracrine signaling mechanisms [19,25]. These stromal cells release factors such as CXCL12, vascular endothelial growth factor (VEGF), and receptor activator of nuclear factor kappa-Β ligand (RANKL). This creates a cytokine environment that supports tumor cell survival, drug resistance, and the formation of angiogenic niches [21,25]. When normal bone remodeling is disrupted, a harmful cycle begins. Tumor-induced osteolysis releases growth factors that encourage cancer cell growth and further bone damage [20,24]. Recent research suggests that the release of calcium ions during osteoclastic resorption may exacerbate this cycle. These ions activate calcium-sensing receptors on tumor cells, leading to increased metastatic growth and resistance to treatment [19,24].

### 2.2. Molecular Pathways

**Figure 2:**
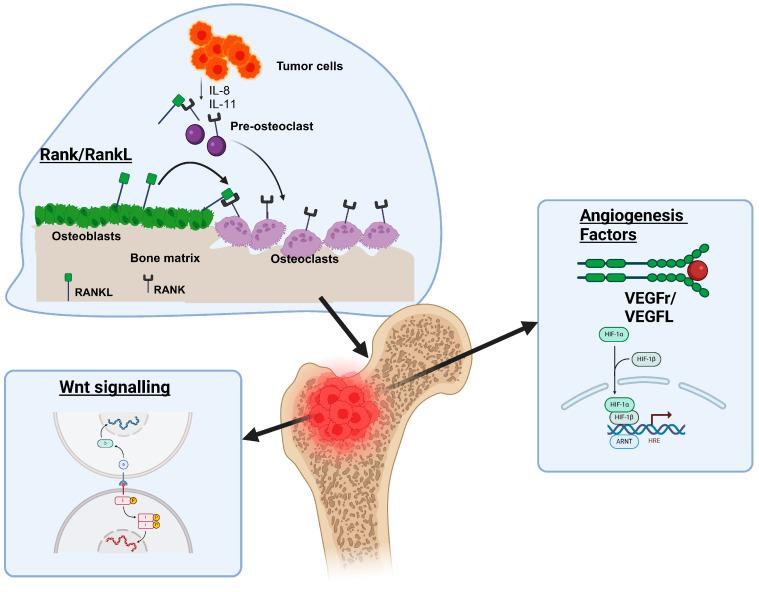
Interactions between tumor cells, bone microenvironment, and angiogenesis in bone metastases: This figure illustrates the complex interplay of cellular and molecular mechanisms involved in bone metastasis. Tumor cells in the bone microenvironment secrete interleukins (IL-8 and IL-11), which stimulate pre-osteoclasts’ maturation into osteoclasts. The receptor activator of nuclear factor kappa-B ligand (RANKL) expressed by osteoblasts binds to RANK on osteoclasts, promoting osteoclast activity and bone resorption. This process is crucial for the remodeling of the bone matrix and the establishment of metastases. Additionally, the Wnt signaling pathway, depicted in the bottom left, influences the regulation of osteoblast and osteoclast functions, critical for maintaining bone homeostasis and metastatic progression. The top right section highlights the role of angiogenesis in supporting tumor growth and metastasis, mediated by vascular endothelial growth factor (VEGF) and its receptors (VEGFRs), regulated through hypoxia-inducible factors (HIF-1α and HIF-1β). These interactions underscore potential biomarkers and therapeutic targets in managing bone metastases.

The section on molecular pathways in bone metastases explores key signaling axes, such as RANK/RANKL/OPG and Wnt signaling, which are critical for understanding how cancers manipulate the bone environment to facilitate metastatic growth. Additionally, it covers the roles of angiogenesis and hypoxia-induced pathways, highlighting their dual functions in bone remodeling and tumor progression. This discussion illuminates the biological interactions at play and underscores potential targets for therapeutic intervention and biomarker development in managing bone metastases (Figure 2).

#### 2.2.1. RANK/RANKL/OPG Axis

The receptor activator of nuclear factor-κB (RANK), along with its ligand RANKL and osteoprotegerin (OPG), is crucial in regulating bone remodeling. Under typical conditions, RANKL is mainly produced by osteoblasts and stromal cells. It binds to RANK on osteoclast precursors, setting off a signaling cascade that promotes the differentiation, activation, and survival of osteoclasts [26]. This balance is countered by OPG, a soluble decoy receptor released by osteoblasts and other mesenchymal cells that competes with RANKL-RANK interaction in a manner that prevents bone resorption [26,27]. The dynamic balance between RANKL and OPG maintains coupled bone resorption and bone formation and ensures the stability of the skeleton [26]. However, when this axis is disrupted, pathological bone deterioration occurs, especially in metastatic cancers that have osteolytic lesions [26,27].

At the molecular level, RANKL binds to RANK, leading to the activation of key pathways, including nuclear factor-κB (NF-κB), mitogen-activated protein kinase (MAPK), and phosphatidylinositol 3-kinase (PI3K)/Akt. These pathways collectively drive osteoclastogenesis and enhance the function of osteoclasts [26,27]. Meanwhile, OPG acts as a natural brake on this mechanism by decreasing the availability of RANKL, thereby regulating bone resorption by osteoclasts [26]. A key factor affecting bone homeostasis is the RANKL/OPG ratio; elevated RANKL or reduced OPG levels lead to increased bone loss [26,27]. Malignant tumor cells exploit this mechanism by secreting cytokines such as prostaglandin E2 (PGE2), interleukin-6 (IL-6), and parathyroid hormone-related peptide (PTHrP). These substances diminish OPG production and enhance RANKL expression in osteoblasts and stromal cells [26,27]. This creates a pro-osteoclastic microenvironment that promotes metastatic colonization.

One key aspect of bone metastases, particularly in breast, prostate, and lung cancers, is the altered RANK/RANKL/OPG axis regulation. Exosomes and various factors released by tumors, such as TGF-β and lncRNA-SOX2OT, convey signals that stimulate osteoclast formation, resulting in osteolysis [26,27]. Consequently, increased osteoclast activity releases matrix-embedded growth factors such as insulin-like growth factor 1 (IGF-1) and bone morphogenetic proteins (BMPs), which drive tumor growth and create a self-reinforcing cycle of bone degradation and metastatic advancement [26]. In non-small-cell lung cancer (NSCLC), clinical studies have shown that higher RANKL:OPG ratios in primary tumors are associated with an elevated risk of skeletal metastasis, emphasizing the prognostic significance of this axis [27].

#### 2.2.2. Wnt Signaling

The Wnt signaling pathway is a vital and conserved molecular cascade significantly influencing embryonic development, tissue maintenance, and cancer progression. It comprises 19 secreted glycoprotein ligands, Frizzled (FZD) receptors, low-density lipoprotein receptor-related proteins 5 and 6 (LRP5/6) co-receptors, and various intracellular effectors. This pathway is categorized into two main types: canonical (β-catenin-dependent) and non-canonical (β-catenin-independent). The canonical pathway is crucial for maintaining bone health, as it influences osteoblast differentiation, bone formation, and skeletal remodeling. In this pathway, Wnt ligands attach to FZD receptors and LRP5/6 co-receptors, stabilizing β-catenin by blocking its phosphorylation and subsequent degradation. Accumulated β-catenin enters the nucleus and activates osteogenic genes such as RUNX2 and osterix by engaging transcription factors like T-cell factor/lymphoid enhancer factor (TCF/LEF) [28,29]. Non-canonical pathways that affect cell migration and calcium flow, including the Wnt/Ca^2+^ and Wnt/planar cell polarity pathways, exhibit a less direct connection to osteogenesis. Proper bone remodeling relies on a careful balance between Wnt activation and inhibition. Disruption of this protein plays a crucial role in various pathological bone disorders, including metastases [28,29,30].

Canonical Wnt signaling is essential for physiological bone remodeling, particularly in the commitment and function of osteoblast lineage. When mesenchymal stem cells (MSCs) are influenced by Wnt ligands, such as Wnt1, Wnt3a, and Wnt10b, they differentiate into osteoprogenitors. These ligands suppress fat and cartilage formation pathways while fostering osteoblast maturation [28,29]. Osteoprotegerin (OPG) levels rise due to stabilized β-catenin, serving as a decoy receptor for RANKL. This action decreases bone resorption and inhibits the formation of osteoclasts. In contrast, Wnt inhibitors like sclerostin (SOST) and Dickkopf-1 (DKK-1) disrupt LRP5/6, destabilizing β-catenin and increasing osteoclast activity. A balance between bone growth and resorption is vital for the stability of the skeletal system. In contrast to mutations that impair LRP5/6 function and cause osteoporosis, transgenic mice overexpressing Wnt10b show increased bone mass due to enhanced osteoblast activity [28,29,30]. This pathway is crucial for communication between osteoblasts and osteoclasts, yet it also has the potential for misuse, being key to maintaining bone homeostasis.

In bone metastases, tumor cells manipulate Wnt signaling to interfere with bone remodeling, leading to either osteolytic or osteoblastic lesions, depending on the type of cancer. Breast and prostate cancers illustrate this complexity. Breast cancer cells frequently release Wnt inhibitors like DKK-1 and SOST. These molecules suppress osteoblast function and encourage osteolysis through unregulated RANKL-mediated osteoclastogenesis [30,31]. Conversely, prostate cancer cells initially produce DKK-1 to enhance osteolytic invasion, but its expression later decreases. This decline allows for a transition to Wnt-driven osteoblasts by activating β-catenin and prolonging RUNX2 expression [32,33]. The bone microenvironment undergoes further disruption due to tumor-derived Wnt ligands, such as Wnt1 and Wnt5a. These ligands induce cancer cells to undergo epithelial–mesenchymal transition (EMT), which enhances their survival and attracts osteoclast precursors. This creates a persistent cycle of bone loss and tumor growth. For instance, Wnt5a activates ROR2 in osteoblasts, promoting JNK signaling and RANKL production, thereby creating a continuous cycle of bone destruction and tumor growth [28,29]. Furthermore, mutations in APC or CTNNB1 (which encodes β-catenin) cause Wnt signaling to be constitutively active in metastatic cells, worsening pathological bone formation in osteoblastic lesions [32,34]. These mechanisms highlight the complexity and context-dependent nature of this pathway’s roles in metastasis.

Therapeutic strategies targeting Wnt signaling are promising for advancing precision medicine in bone metastases. Researchers are examining biomarkers, including serum DKK-1, sclerostin, and Wnt ligand levels, to help predict metastatic progression and treatment outcomes. High levels of DKK-1 are associated with osteolytic damage in multiple myeloma and breast cancer, whereas a reduction in sclerostin is linked to osteoblastic repair in prostate cancer [31,32]. Clinical trials evaluate pharmacological inhibitors like romosozumab for sclerostin and BHQ880 for DKK-1 to improve bone integrity via Wnt-driven osteogenesis [29,30]. Monoclonal antibodies, such as vantictumab, that target FZD receptors and small-molecule β-catenin inhibitors like PRI-724 are designed to inhibit oncogenic Wnt activation in tumors. Nonetheless, systemic toxicity remains a significant concern. To address this, researchers are creating advanced nanotherapeutic platforms, including LRP5-targeted nanoparticles, which aim to deliver Wnt modulators directly to metastatic sites. This strategy seeks to diminish off-target effects [29,34]. These innovations highlight the potential of Wnt pathway modulation to effectively manage skeletal complications and tumor growth, thereby advancing the concept of precision oncology in bone metastases.

#### 2.2.3. Angiogenesis and Hypoxia-Induced Pathways

Angiogenesis, the process of forming new blood vessels from existing ones, plays a crucial role in promoting tumor growth and facilitating cancer’s spread to bone [35]. Metastatic cells in the skeletal microenvironment produce vascular endothelial growth factor (VEGF) and other pro-angiogenic substances. This stimulates the proliferation of endothelial cells and leads to the formation of immature vascular networks that support tumor survival and growth [36]. These new blood vessels often exhibit structural irregularities, including insufficient pericyte coverage and abnormal branching. Such defects increase hypoxia while also providing metabolic support to growing metastatic lesions [35]. In addition to VEGF, factors such as placental growth factor (PlGF) and angiopoietin-2 (Ang-2) enhance angiogenic signaling by influencing endothelial cell migration and vascular permeability. This establishes a positive feedback loop that promotes metastasis within the bone marrow [36]. Research using preclinical models indicates that therapies targeting both VEGF and platelet-derived growth factor (PDGF) pathways can effectively reduce bone destruction and tumor-induced changes in vascular structures, emphasizing the critical role of angiogenesis in the pathology of bone metastasis [36].

The hypoxic bone marrow niche stimulates the activation of hypoxia-inducible factors (HIF-1α and HIF-2α). These factors are crucial for transcriptional reprogramming, enabling tumor cells to adapt to their environment through glycolytic metabolism, and release pro-angiogenic cytokines [30,35]. Under conditions of low oxygen, HIF-α subunits avoid proteasomal degradation, dimerize with HIF-1β, and bind to hypoxia-response elements (HREs) to enhance the expression of genes essential for metastatic survival, such as carbonic anhydrase IX (CAIX) and glucose transporter 1 (GLUT1) [37]. This metabolic transition enables tumor cells to survive in oxygen-deficient regions while secreting extracellular vesicles loaded with HIF-dependent miRNAs, preparing the bone microenvironment for metastatic colonization [35]. Notably, breast cancer cells lacking estrogen receptors (ERs) show elevated HIF activity, which is linked to an increase in osteolytic lesion formation and rapid bone destruction in preclinical studies [37].

Targeting angiogenesis and hypoxia pathways has led to the development of effective clinical strategies for treating bone metastases. Bevacizumab, a monoclonal antibody targeting VEGF-A, shows promise in reducing skeletal tumor load and preventing bone damage associated with angiogenesis. This is especially true when paired with tyrosine kinase inhibitors in animal models [36]. Emerging hypoxia-targeted treatments, such as the HIF inhibitor 2-methoxyestradiol, effectively slow tumor progression and inhibit osteoclast differentiation in ER-negative breast cancer bone metastases, offering dual therapeutic benefits [37]. Radiotheranostic approaches that incorporate hypoxia-activated prodrugs (HAPs) and PSMA-targeting radioligands enable accurate delivery of cytotoxic agents to metastatic sites while safeguarding healthy bone marrow [38]. Serum VEGF and urinary N-telopeptide (uNTx) when continuously monitored serve as non-invasive biomarkers to evaluate treatment response and anticipate skeletal-related events, aiding personalized therapy adjustments [35]. Ongoing clinical trials are investigating the combination of HIF inhibitors and immune checkpoint blockers to counter hypoxia-induced immunosuppression and improve T-cell infiltration in metastatic bone lesions [37].

#### 2.2.4. Clinical Implications

The bone microenvironment experiences notable alterations during metastatic colonization, characterized by an imbalance between osteoclastic bone resorption and osteoblastic bone formation. This disruption results in measurable biomarkers that hold diagnostic significance [39,40]. Osteolytic lesions emerge from tumor-induced osteoclast activation, creating a relentless cycle of bone damage and tumor growth. Additionally, decreased osteoblast activity compromises the skeletal integrity [39,41]. These molecular and cellular changes facilitate disease progression while also generating systemic biomarkers that reflect ongoing pathological activity, thereby supporting the early detection and long-term monitoring of bone metastases [40,41].

Recent insights into the molecular mechanisms underlying bone metastasis have led to the development of targeted therapies that disrupt the harmful interactions between tumors and bone. Bisphosphonates, such as zoledronic acid, promote osteoclast apoptosis by inhibiting farnesyl pyrophosphate synthase, thereby reducing bone resorption and related skeletal complications [42]. Similarly, denosumab, a monoclonal antibody that targets RANKL, prevents RANKL from binding to its receptor on osteoclast precursors. This action effectively suppresses osteoclast differentiation and interrupts osteolytic progression [39]. New approaches, such as combining bisphosphonates with low-intensity pulsed ultrasound, enhance drug penetration into metastatic areas, thereby improving anti-tumor effects while reducing systemic toxicity [43]. These therapies not only help stabilize skeletal structure but also indirectly hinder tumor survival by depriving cancer cells of growth factors released during bone resorption, including TGF-β and IGF-1 [39,41].

Pathological bone remodeling is a crucial biomarker for assessing the aggressiveness of the disease and predicting patient outcomes. High baseline levels of TRACP-5b and BALP suggest an increased risk of skeletal-related events, such as pathological fractures and spinal cord compression. A quick increase in biomarkers following treatment often indicates resistance to therapy [39,41]. In breast cancer, for example, lasting reductions in NTx levels after denosumab treatment correlate with longer intervals before skeletal complications occur and improved survival rates [39]. Including biomarker dynamics in clinical algorithms aids in risk stratification, guiding decisions on whether to intensify treatment or pursue palliative care. Furthermore, liquid biopsy markers, such as circulating tumor cells (CTCs) and exosomal miRNAs, emerge as critical prognostic tools. They offer insights into tumor diversity and metastatic capabilities [41,44]. By combining biomarker information with clinical and imaging data, healthcare providers can tailor treatment strategies to individual disease traits, enhancing the precision medicine approach for metastatic bone disease [39,40].

## 3. Biomarkers in Bone Metastases

### 3.1. Introduction to Biomarkers

Bone metastases are a common and serious problem for people with advanced cancers, particularly breast and prostate cancer [45]. When cancer spreads to the bones, it can result in painful complications like fractures, spinal cord issues, and increased calcium levels. These problems can greatly affect a person’s well-being and daily life [45]. To aid detection and management, biomarkers have been identified as key disease progression and treatment response indicators. A biomarker is a measurable indicator of a biological state or condition that plays a crucial role in disease detection and management. In clinical oncology, biomarkers provide critical insights into cancer progression, therapeutic response, and disease recurrence, aiding in precision medicine [46]. By providing molecular and biochemical insights, biomarkers can complement imaging and clinical evaluation to improve the detection of occult bone lesions, stratify patients by risk, guide targeted therapy selection, and monitor disease activity in real time [45,47]. In essence, biomarkers related to bone metastasis serve several crucial purposes. They help detect metastatic disease, estimate the progression of the disease, suggest how patients might respond to specific treatments, and monitor the effectiveness of those treatments. By using these markers, doctors can personalize cancer care, focusing on high-risk patients who may benefit from preventive bone-targeted therapy [45].

### 3.2. Classification of Biomarkers

**Figure 3:**
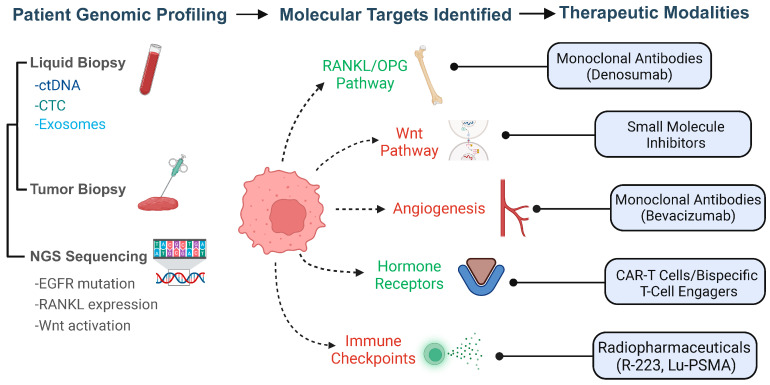
Integration of patient genomic profiling and therapeutic targeting in bone metastasis: This figure outlines the process of patient genomic profiling leading to personalized therapeutic interventions for bone metastasis. It begins with the collection of genetic material through liquid biopsy (ctDNA, CTCs, exosomes) and tumor biopsy, followed by next-generation sequencing (NGS), which identifies specific mutations and pathway activations such as EGFR mutations, RANKL expression, and Wnt pathway activation. Identified molecular targets include the RANKL/OPG pathway, Wnt signaling, angiogenesis pathways, hormone receptors, and immune checkpoints. These targets correspond to various therapeutic modalities, including monoclonal antibodies (e.g., Denosumab for RANKL, Bevacizumab for angiogenesis), small-molecule inhibitors, CAR-T cells and bispecific T-cell engagers for immune modulation, and radiopharmaceuticals (e.g., R-223, Lu-PSMA). This strategic approach underscores the potential for precision medicine in treating bone metastases by tailoring therapies based on specific genetic profiles and molecular characteristics.

In oncology, biomarkers are sorted by the specific questions they help address. This sorting is vital for their effective use in tailored medicine. For bone metastasis, biomarkers are mainly divided into four types: diagnostic, prognostic, predictive, and monitoring (Figure 3).

Diagnostic biomarkers indicate the presence of disease, helping to detect or confirm bone metastases, and are used to screen or identify metastasis in patients (for example, a blood marker that suggests metastatic spread to the bone) [48]. Additionally, diagnostic biomarkers are used to detect the presence of bone metastases, thereby distinguishing metastatic involvement from primary bone malignancies. These biomarkers help identify skeletal metastases at an early stage, improving treatment planning and patient outcomes [49].

Prognostic biomarkers provide insights into how a disease may progress or what the potential outcomes might be, regardless of treatment. They can help forecast the chances of disease worsening or the overall survival probability [50]. They help in assessing disease progression and survival outcomes, thereby assisting clinicians in risk stratification and individualized therapeutic decision-making [51].

Predictive biomarkers forecast a patient’s response to specific therapies by identifying who will likely benefit from a given treatment or who may not. This information is critical in guiding therapy selection, such as in the case of bone-targeted treatments or systemic anticancer drugs [50]. They minimize unnecessary exposure to ineffective therapies by predicting treatment response [52].

Monitoring biomarkers are measured serially over time to indicate the status of the disease and the effectiveness of treatments. They help observe how the disease changes, show whether a therapy is having an effect or if cancer is worsening, and can also identify recurrence in bone metastasis. Regularly evaluating these biomarkers allows for timely adjustments in treatment, improving overall patient outcomes [35].

Identifying these categories is important because a marker useful for diagnosis might not predict how a disease will progress, and a marker showing an aggressive disease does not always reveal how someone will respond to a certain medication [53]. Misclassification can lead to suboptimal treatment choices, whereas accurate classification enables doctors to incorporate biomarkers into their decision-making process. Notably, some biomarkers even have overlapping functions, which can complicate these distinctions [53].

### 3.3. Diagnostic Biomarkers

#### 3.3.1. Circulating Tumor Cells (CTCs)

Circulating tumor cells (CTCs) originate from primary or metastatic tumors and migrate into the peripheral bloodstream. These cells act as potential indicators for the diagnosis and tracking of bone metastases in different types of cancer, such as breast, prostate, lung, and neuroendocrine tumors. Persistently high levels of CTCs are linked to worse survival outcomes, with baseline CTC-negative patients obtaining a significantly longer OS than CTC-positive patients [54].

Circulating tumor cells (CTCs) have been extensively studied for their role in identifying bone metastases. In individuals with breast cancer, those with bone metastases usually exhibit significantly higher CTC levels than those without, and the number of CTCs has been associated with the progression of the disease [55]. CTCs can be divided into three categories, epithelial, mesenchymal, and hybrid epithelial–mesenchymal types, with mesenchymal CTCs displaying increased migratory and invasive abilities [55]. Additionally, CTC clusters, which are groups of two or more tumor cells, are more likely to lead to metastatic progression than individual CTCs [55].

Studies on circulating tumor cells (CTCs) in lung cancer indicated a significant link between the presence of bone metastases and CTCs [56]. Similarly, the detection of CTCs in neuroendocrine tumors (NETs) demonstrated a strong association with the onset of bone metastases, implying that they could act as prognostic indicators [56]. In breast cancer patients, researchers have observed a connection between CTC detection and FDG-PET/CT findings, a commonly used imaging method for evaluating bone metastases [56]. This suggests that CTCs could enhance diagnostic accuracy when combined with imaging techniques. Moreover, higher CTC levels have been associated with worse survival outcomes, indicating their prognostic value [55].

Various molecular markers on circulating tumor cells (CTCs) have been investigated in relation to bone metastases. The chemokine receptor CXCR4 is recognized for facilitating the migration of tumor cells to bone and is commonly overexpressed in CTCs from patients with bone metastases [56]. Increased expression levels of CXCR4 have been connected to greater osteotropism in tumor cells, suggesting its potential as a predictive biomarker [56]. Furthermore, other surface markers such as CD44 and CD74 have been linked to bone metastasis and could function as potential biomarkers for circulating tumor cells (CTCs) [41].

**Figure 4:**
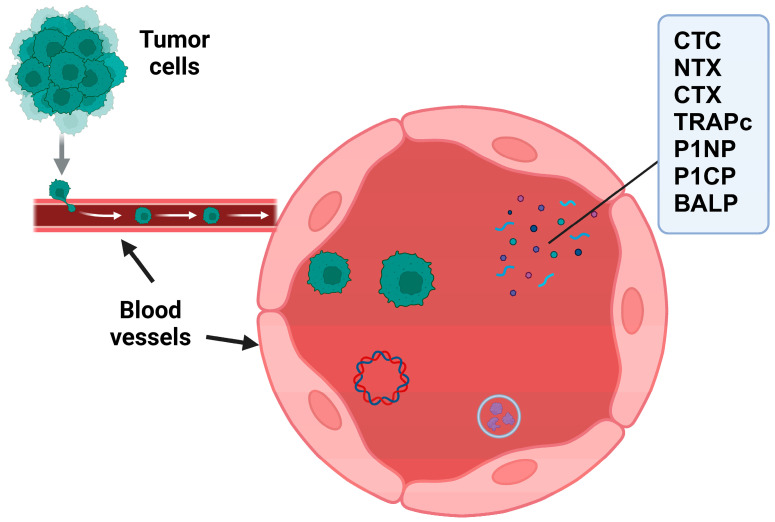
Circulating tumor cells (CTCs) and biomarkers of bone remodeling in the context of metastasis: This diagram depicts the dissemination of tumor cells from the primary site into the bloodstream, where they become circulating tumor cells (CTCs). It highlights the interaction between CTCs and the bone environment, where they can contribute to metastatic bone disease. The right-hand panel lists key biomarkers associated with bone remodeling and metastasis: C-terminal telopeptide (CTX) and N-terminal telopeptide (NTX) for bone resorption, tartrate-resistant acid phosphatase (TRAPc), procollagen type I N propeptide (P1NP), procollagen type I C propeptide (P1CP), and bone-specific alkaline phosphatase (BALP). These biomarkers serve as crucial indicators for the diagnosis, monitoring, and understanding of the metastatic process, as well as potential therapeutic targets.

The prognostic value of circulating tumor cells (CTCs) has been confirmed in various studies. For instance, in prostate cancer cases, increased CTC counts were associated with a higher risk of developing bone metastases and decreased survival rates [40]. The existence of circulating tumor cells (CTCs) in neuroendocrine tumors (NETs) has been associated with lower survival rates, underscoring their importance as a crucial prognostic marker [56]. However, multiple challenges are related to the detection and analysis of CTCs. The heterogeneity of CTCs, their limited presence in the bloodstream, and variations in detection methods obstruct their wider use in clinical applications. The CellSearch system, a conventional technique for isolating CTCs, utilizes enrichment based on epithelial cell adhesion molecule (EpCAM), which may not be able to identify all CTC subpopulations, especially those undergoing the epithelial–mesenchymal transition (EMT) [56]. Furthermore, certain patients with bone metastases may have undetectable CTC levels, indicating that the presence of CTCs alone does not necessarily indicate disease progression with great sensitivity, according to studies [56]. Techniques such as liquid biopsies which combine CTC detection with other circulating biomarkers (Figure 4), including circulating tumor DNA (ctDNA) and exosomal markers, could enhance the sensitivity and specificity of diagnostics based on CTCs [41].

#### 3.3.2. Bone Turnover Markers

Bone turnover markers (BTMs) are biochemical indicators that reflect the bone remodeling process, correlating with osteoblasts’ and osteoclast functions. These markers are typically categorized into two main types: those that signify bone formation and those that signify bone resorption. Extensive studies have been performed on these markers for their potential use in diagnosing and predicting the progression of bone metastases in different cancers, such as breast, prostate, lung, and renal cancers.

Osteoblasts primarily produce markers of bone formation and include bone-specific alkaline phosphatase (BALP), procollagen type 1 N-terminal propeptide (P1NP), procollagen type 1 C-terminal propeptide (P1CP), and osteocalcin.

BALP has become a crucial marker for identifying and predicting the progression of bone metastases. Elevated BALP levels are associated with osteoblastic activity, which is particularly relevant in prostate cancer, where bone metastases generally display osteoblastic features [55]. Studies indicate that individuals with bone metastases have significantly higher BALP levels compared to those without, and its diagnostic accuracy is comparable to imaging techniques like bone scintigraphy [57].

P1NP, a collagen synthesis marker, is another important bone formation marker. It has been utilized to identify bone metastases in patients with breast and prostate cancer [55]. Elevated levels of P1NP suggest heightened osteoblastic activity, frequently seen in metastatic bone lesions. Nevertheless, its specificity is somewhat limited, as P1NP levels may also rise in other conditions impacting bone metabolism, like osteoporosis [40].

Osteocalcin, a non-collagenous protein produced by osteoblasts, has been studied for its role in bone metastases. However, its effectiveness as a diagnostic tool is constrained due to variations influenced by systemic factors unrelated to bone metastases [58]. While osteocalcin is valuable for assessing bone turnover, it lacks the specificity needed to serve as an independent biomarker for bone metastases.

Markers of bone resorption provide insight into bone breakdown by osteoclasts and include indicators like C-terminal telopeptide of type I collagen (CTX), N-terminal telopeptide of type I collagen (NTX), deoxypyridinoline (DPD), and tartrate-resistant acid phosphatase 5b (TRACP-5b).

CTX and NTX, which reflect the breakdown of type I collagen, are commonly used to evaluate bone metastases. NTX, in particular, has shown significant prognostic value in patients with bone metastases originating from breast and prostate cancers. Elevated NTX levels correlate with the severity of bone lesions and can anticipate occurrences related to skeletal complications. Studies involving breast cancer patients with bone metastases indicated that NTX levels were significantly higher in those with progressive disease compared to individuals with stable metastases [58].

DPD, a peptide linked to collagen cross-linking, acts as another biomarker for bone resorption. Increased urinary DPD levels have been observed in patients with bone metastases from lung, breast, and prostate cancers [58]. However, its effectiveness is somewhat limited due to fluctuations in urinary excretion rates, which may lead to variable results.

TRACP-5b, an enzyme produced by activated osteoclasts, has emerged as a key indicator for bone metastases. In contrast to other resorption markers, TRACP-5b is largely independent of kidney function, which enhances its reliability as a measurement [57]. Research has demonstrated that patients with prostate cancer who have bone metastases exhibit higher levels of TRACP-5b, which correlate with disease progression and overall survival outcomes [40].

While BTMs provide valuable insights regarding bone metastases, their clinical utility is limited by fluctuations caused by physiological bone turnover, hormonal influences, and constraints in assay methodologies [55]. Additionally, BTMs may be influenced by conditions such as osteoporosis, fractures, and metabolic bone disorders, making them not solely indicative of cancer-related bone metastasis [58]. 

The precision of diagnosis improves when various bone turnover markers (BTMs) are used in conjunction with imaging techniques. For instance, research has shown that integrating NTX and BALP levels with PET/CT or bone scintigraphy facilitates the early detection of bone metastases [57]. Furthermore, for patients undergoing treatment with bone-targeting agents, such as bisphosphonates, regular monitoring of bone turnover markers (BTMs) can help predict skeletal complications and assess treatment effectiveness [40].

#### 3.3.3. Imaging-Based Biomarkers

**Figure 5:**
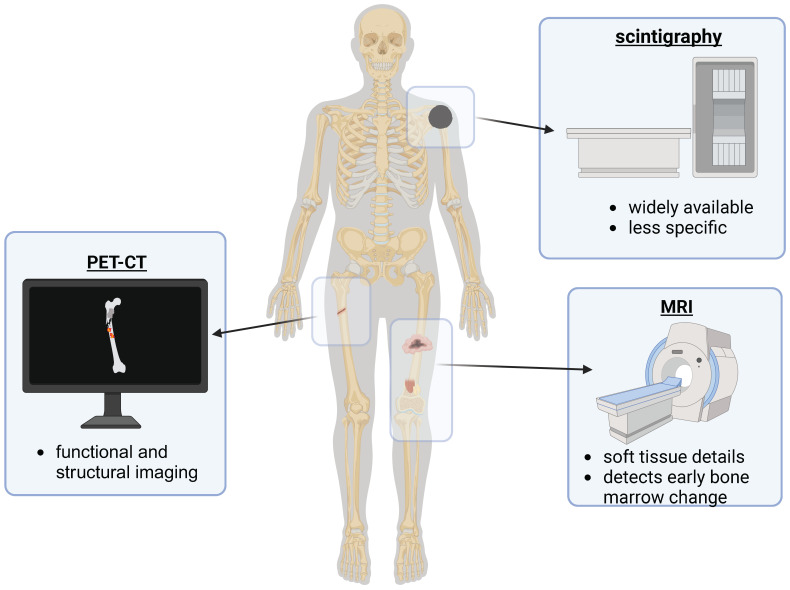
Diagnostic imaging modalities for bone metastasis detection. This figure provides an overview of the primary imaging techniques used in the diagnosis and monitoring of bone metastases. Each modality is linked to specific features and uses within the clinical context: PET-CT combines positron emission tomography (PET) and computed tomography (CT) to offer both functional and structural imaging. This modality is crucial for identifying active metastatic sites and assessing metabolic activity. Scintigraphy: A widely available nuclear medicine test that uses radioactive substances to detect abnormalities in bone metabolism, though it is less specific compared to other modalities. MRI: Magnetic resonance imaging provides detailed images of soft tissues and is highly sensitive for detecting early changes in the bone marrow associated with metastasis. These imaging techniques are integral to the comprehensive assessment of bone metastases, aiding in the precise localization of lesions and guiding subsequent therapeutic strategies.

Imaging biomarkers have become integral tools for diagnosing bone metastases, monitoring treatment efficacy, and predicting disease progression. Numerous imaging techniques, including bone scintigraphy, magnetic resonance imaging (MRI), computed tomography (CT), and positron emission tomography (PET), have been assessed for their ability to accurately diagnose metastatic bone lesions and their applicability in clinical settings (Figure 5).

##### Positron Emission Tomography (PET) and PET/CT

PET imaging, particularly when combined with CT (PET/CT), has revolutionized the detection of bone metastases by providing both functional and structural information. The primary radiotracers utilized in PET imaging are ^18^F-fluorodeoxyglucose (^18^F-FDG), ^18^F-sodium fluoride (^18^F-NaF), and ^68^Ga-prostate-specific membrane antigen (^68^Ga-PSMA).

^18^F-FDG PET/CT has been extensively employed for the detection of bone metastases, particularly among patients with breast cancer. A meta-analysis comparing ^18^F-FDG PET/CT to bone scintigraphy in patients with breast cancer revealed that PET/CT had a notably higher sensitivity (93%) and specificity (99%) in comparison to bone scintigraphy, which exhibited a sensitivity of 81% and specificity of 96% [59]. These results suggest that ^18^F-FDG PET/CT serves as a more effective diagnostic approach for identifying bone metastases in patients with breast cancer.

In prostate cancer cases, ^68^Ga-PSMA PET/CT has displayed exceptional accuracy in spotting bone metastases. A prospective study that compared ^68^Ga-PSMA PET/CT, ^18^F-NaF PET/CT, and whole-body MRI revealed that PSMA-PET/CT achieved the highest sensitivity (100%) and specificity (100%), outperforming both ^18^F-NaF PET/CT and MRI [60]. This suggests that PSMA-PET/CT should be regarded as the gold standard for identifying bone metastases in patients with prostate cancer.

##### Computed Tomography (CT)

CT imaging is extensively utilized to identify structural alterations in bone metastases, such as destruction of the cortex, sclerosis, and pathological fractures. Although CT offers remarkable anatomical detail, it falls short in identifying early metastatic involvement prior to substantial structural damage. Its efficacy is enhanced when used in conjunction with PET, especially in PET/CT, which enables the integration of functional and structural data for a more accurate diagnosis [61].

##### Magnetic Resonance Imaging (MRI)

MRI is notably skilled at detecting early metastatic changes in the bone marrow before significant bone damage occurs. Whole-body MRI, including diffusion-weighted imaging (DWI), has become increasingly popular for staging in prostate and breast cancers. In a prospective investigation comparing ^68^Ga-PSMA PET/CT, NaF-PET/CT, and whole-body MRI in patients with prostate cancer, MRI exhibited the lowest sensitivity (80%) and specificity (83%) among the three methods, making it less advantageous than PET-driven techniques for detecting bone metastases [60].

##### Bone Scintigraphy

Bone scintigraphy with technetium-99m methylene diphosphonate (^99m^Tc-MDP) remains one of the most frequently used imaging methods for detecting bone metastases. It is highly sensitive in recognizing osteoblastic activity, but it lacks specificity, as heightened uptake can also be seen in benign bone conditions [60]. Despite its widespread use, PET/CT and MRI are increasingly supplanting bone scintigraphy due to their greater accuracy and ability to identify early metastatic changes.

##### Clinical Utility and Limitations

While imaging-based biomarkers have greatly enhanced the detection and monitoring of bone metastases, each imaging technique has its drawbacks. PET/CT using PSMA tracers is the most accurate technique for detecting metastases in prostate cancer, although it is costly and not readily accessible. MRI provides excellent soft tissue differentiation but is limited in identifying metastatic bone lesions specifically. Bone scintigraphy is a more affordable option, yet it is less accurate compared to advanced imaging methods. Combining various imaging modalities, like PET/CT with MRI, can enhance diagnostic accuracy and improve patient management.

### 3.4. Prognostic Biomarkers

#### 3.4.1. Circulating DNA (ctDNA)

Broken-off DNA from tumors can enter the bloodstream from the active secretion of cancer cells, necrosis, or apoptosis. This broken DNA is referred to as circulating tumor DNA (ctDNA). CtDNA provides a non-invasive method for evaluating tumor burden, identifying minimal residual disease (MRD), and predicting prognosis in patients with bone metastases. Research investigating ctDNA as a predictive biomarker has concentrated on its levels, genetic mutations, and alignment with tissue-based profiling across various cancers.

Numerous studies have assessed the levels of ctDNA and their prognostic significance in various malignancies associated with bone metastases. Standard techniques for measuring ctDNA and pinpointing genetic mutations include droplet digital PCR (ddPCR) and next-generation sequencing (NGS) [62,63]. CtDNA levels were 1.4 times higher in NSCLC patients with bone metastases than in those without, demonstrating a link between ctDNA concentration and metastatic spread [63]. Meanwhile, ctDNA-positive status was detected in 62.1% of patients with triple-negative breast cancer (TNBC), with higher detection rates seen in hormone receptor-negative cases [62]. However, different tumors exhibit varying sensitivities for ctDNA detection. Compared to tissue-based molecular profiling, ctDNA KRAS mutations were detected with 80.6% sensitivity and 100% specificity in a cohort of 186 NSCLC patients [64]. The reliability of ctDNA as a single biomarker was limited by false-negative results that occurred in instances with minimal tumor load [63].

In breast cancer, the most commonly found gene mutations in ctDNA were TP53 (34%), BRCA1 (20%), and BRCA2 (17%) [64]. Recurrence-free survival (RFS) was considerably lower in patients with TP53 and/or BRCA1 mutations, especially in TNBC. Early ctDNA screening before treatment enabled the detection of high-risk patients, allowing for personalized therapeutic approaches. However, the rates of ctDNA positivity were lower among patients with luminal breast cancer, limiting its prognostic relevance in this particular subgroup [62].

A hazard ratio (HR) of 1.63 in patients with non-small-cell lung cancer (NSCLC) with bone metastases compared to those without metastasis indicated that patients in the former group had a worse overall survival (OS), with KRAS and EGFR mutations in circulating tumor DNA (ctDNA) being linked to this poorer outcome [63]. There was a 94.7% agreement between ctDNA and tumor tissue for KRAS and EGFR mutations, reinforcing the reliability of ctDNA for molecular profiling [63]. Additionally, 10% of NSCLC patients with bone metastases exhibited mutations in EGFR (T790M), indicating the development of resistance to targeted forms of therapy. This underscores the value of using ctDNA to monitor disease progression [63].

While there is a high probability of bone metastases occurring in prostate cancer (65–75%), detecting ctDNA in this scenario is challenging because osteoblastic lesions release lower amounts of DNA [65]. Nevertheless, mutations in TP53 and AR (androgen receptor) present in ctDNA were associated with poor survival outcomes and resistance to androgen-deprivation therapy (ADT) [65]. The levels of ctDNA found in prostate cancer were lower than those observed in NSCLC and breast cancer, affecting the prognostic value and detection rates [65].

Consistently higher ctDNA levels were linked to worse survival outcomes across multiple studies, suggesting its potential as a tool for risk stratification [65]. The notable reduction in OS highlights the significant impact on prognosis in NSCLC patients with high frequencies of KRAS mutant alleles [63]. TNBC patients with mutations in BRCA1 and TP53 experienced worse relapse-free survival (RFS) in breast cancer, illustrating the importance of mutation-specific ctDNA profiling for prognostic evaluation [62].

Some Patients Have Low ctDNA Levels: Reduced ctDNA shedding is seen in prostate cancer and early-stage metastatic cases, which reduces the sensitivity of detection [65]. Lack of Standardized Thresholds: Clinical application and cross-study comparisons are made more difficult by the absence of standardized cutoff values for determining ctDNA positivity [64]. Background Noise and False Negatives: Especially in individuals with minimal tumor burden, normal cell-free DNA (cfDNA) may obstruct ctDNA detection, potentially resulting in false negatives [63]. Variable Concordance with Tumor Tissue: Although NSCLC exhibited 94.7% agreement, ctDNA–tissue concordance varied for other malignancies, such as the breast and prostate, necessitating additional testing [62,63,65].

#### 3.4.2. Bone Matrix Proteins

Bone matrix proteins play a crucial role in the prognosis of bone metastases in various types of cancer. These proteins regulate bone remodeling processes and can result in osteoblastic or osteolytic lesions, depending on the type of cancer. Several bone matrix proteins have been identified as predictive biomarkers, including osteopontin (OPN), bone sialoprotein (BSP), matrix metalloproteinases (MMPs), transforming growth factor-beta (TGF-β), interleukin-11 (IL-11), and connective tissue growth factor (CTGF). CTGF causes angiogenesis, migration, and cell adhesion. Increased CTGF expression has been linked to bone metastases in patients with hepatocellular carcinoma (HCC), suggesting that it may serve as a valuable biomarker. Immunohistochemical studies on curative resection HCC patients revealed a substantial link between high levels of CTGF expression in tumor cells and the risk of bone metastases [66]. Likewise, a critical indicator for the prognosis of bone metastases in HCC is IL-11, a cytokine implicated in osteoclastogenesis. A poor prognosis and higher osteolytic activity are linked to high IL-11 expression [66]. For the development of bone metastases in HCC, peritumoral expression of MMP-1, a matrix metalloproteinase involved in extracellular matrix degradation, has also been found to be an independent predictor [66].

Various malignancies, including breast, prostate, and lung cancers, have been linked to bone metastases through the glycoprotein osteopontin (OPN) released by osteoblasts. OPN facilitates tumor cell migration and adherence to the bone matrix, thereby promoting cancer spread. Patients with bone metastases from prostate cancer (PCa) and non-small-cell lung cancer (NSCLC) have been found to have elevated serum OPN levels, which are associated with poorer survival outcomes [40,52]. Furthermore, a higher risk of bone metastases in patients with lung cancer has been linked to single-nucleotide polymorphisms in the OPN promoter region [52].

Bone sialoprotein (BSP), a non-collagenous bone matrix protein, has been studied in patients with lung cancer and bone metastases. Increased BSP expression in cancer cells is correlated with an increased likelihood of bone metastases, making it a valuable tool in identifying high-risk patients [40]. Furthermore, the degree of bone metastases in NSCLC has been linked to elevated serum BSP levels, indicating the potential utility of this biomarker for early diagnosis [40].

Biomarkers for bone production and remodeling include peptides derived from collagen type I, such as the pro-collagen type I N-terminal propeptide (P1NP) and the pro-collagen type I C-terminal propeptide (P1CP). In prostate cancer patients with bone metastases, increased serum levels of PINP and PICP have been observed; PINP has been found to have high specificity as well as sensitivity in identifying early metastatic occurrences [58]. Moreover, higher PINP levels have been linked to shorter survival times for patients with metastatic prostate cancer [58].

While these bone matrix proteins provide valuable prognostic insights, their clinical applicability is influenced by the type of disease and the degree of metastasis. OPN and BSP are particularly helpful for assessing bone metastases in lung and prostate cancers, whereas other markers such as CTGF, IL-11, and MMP-1 have demonstrated greater prognostic significance in hepatocellular carcinoma (HCC). To evaluate the progression of bone metastases in prostate cancer, PINP, PICP, and BALP are essential. However, there remain challenges in standardizing and validating these biomarkers through extensive clinical trials to enhance their reliability for routine clinical use.

#### 3.4.3. LncRNAs

LncRNAs play a vital role in regulating gene expression and the progression of metastases, making them significant prognostic markers in cases of bone metastases. In patients with metastatic breast and prostate cancers, higher levels of specific lncRNAs, such as MALAT1 and HOTAIR, have been correlated with poorer outcomes. In breast cancer, the long non-coding RNA ANCR has been connected to bone degradation, enhancing the invasiveness of metastatic cells. Likewise, studies have shown that lncRNA ZEB1-AS1 contributes to a higher risk of bone metastases in non-small-cell lung cancer (NSCLC) by facilitating the epithelial-to-mesenchymal transition (EMT), which enables cancer spread. However, challenges persist in reliably detecting and stabilizing lncRNAs in clinical samples [67].

### 3.5. Predictive Biomarkers

#### 3.5.1. Response to Bone-Targeted Therapies as Predictive Biomarkers in Bone Metastasis

Bone-targeting treatments, primarily bisphosphonates and RANKL inhibitors, are frequently used to manage bone metastases in various cancers, including breast and prostate cancer. Their impact on altering the bone microenvironment and diminishing skeletal-related events (SREs) has prompted research into their possible role as predictive biomarkers. The ability of these therapies to influence disease advancement, response strategies, and metastatic burden establishes a basis for evaluating their predictive significance in patient management.

Research has examined the role of bisphosphonates as predictive biomarkers, focusing on their impact on disease progression and bone-related problems. In breast cancer, bisphosphonates have demonstrated a capacity to reduce bone metastases and mortality in postmenopausal women, suggesting a potential stratification role connected to menopausal status and tumor characteristics [68]. Additionally, the transcription factor mesenchymal aponeurotic fibrosarcoma (MAF) has been identified as a potential biomarker for predicting responses to bisphosphonates, as patients showing normal MAF expression experience the greatest benefits from the treatment [68].

Nonetheless, there are constraints related to bisphosphonates as predictive biomarkers. Different cancer types show varying responses; for instance, prostate and lung cancers do not exhibit the same degree of metastasis prevention [68]. Moreover, different dosing frequencies have been assessed, indicating that although standard administration (every 4 weeks) remains effective, de-escalation protocols (every 12 weeks) may provide similar advantages with reduced adverse effects [69]. These results suggest that although the response to bisphosphonates offers crucial prognostic information, more molecular markers are needed for accurate patient selection.

Denosumab demonstrated its effectiveness relative to bisphosphonates in delaying SREs in individuals with castration-resistant prostate cancer, highlighting its potential predictive value [70]. Additionally, the differing efficacy of denosumab across various cancer types suggests its possible predictive role in identifying patients who are at a heightened risk for skeletal complications [35].

Despite these advantages, concerns remain regarding the specificity of RANKL inhibitors as predictive biomarkers. Denosumab’s effects on metastasis suppression vary, unlike bisphosphonates; some studies suggest it has no discernible impact on overall survival [68]. Furthermore, although it successfully reduces SREs, research is still ongoing to determine its capacity to forecast the long-term course of the disease [35]. Its predictive ability may, therefore, be enhanced by continuing research on combinatorial biomarker techniques that combine immune profiling and bone turnover markers. Breast and prostate cancers, which are major causes of bone metastases, display unique osteolytic and osteoblastic patterns, requiring customized predictive approaches. Incorporating liquid biopsy techniques like circulating tumor DNA (ctDNA) and bone turnover markers might enhance predictive precision [40]. Moreover, advancements in imaging methods, such as multimodal imaging, offer promising avenues for improving the assessment of treatment responses [71].

#### 3.5.2. Biomarkers for Immunotherapy Response in Bone Metastases

The introduction of immune checkpoint inhibitors (ICIs) has transformed cancer therapy, providing significant advantages for many cancers, especially those susceptible to bone metastases, including breast, prostate, lung, kidney, and gastric cancers [72,73,74]. Nonetheless, the differences in patient responses have highlighted the importance of identifying predictive biomarkers to categorize patients according to their potential to gain advantages from immunotherapy [72,73]. This section explores the potential and challenges of predicting biomarkers associated with the outcomes of immunotherapy in patients with bone metastases.

By targeting cytotoxic T-lymphocyte-associated protein 4 (CTLA-4), programmed cell death protein 1 (PD-1), and programmed death-ligand 1 (PD-L1), immune checkpoint drugs have shown efficacy against various tumor types. Numerous studies on PD-L1 expression as a predictive biomarker have revealed that improved response rates in kidney cancer, melanoma, and non-small-cell lung cancer (NSCLC) are associated with higher PD-L1 levels [73]. However, because T-cell infiltration is obstructed by the immunosuppressive properties of the bone microenvironment, the prognostic value of bone metastases remains contested [74]. Tumor mutational burden (TMB) has been investigated as a potential biomarker, with studies suggesting that tumors exhibiting higher TMBs demonstrate better responses to ICIs [39]. However, bone metastases typically show a lower TMB than other metastatic sites, which may limit their predictive significance [39]. Furthermore, microsatellite instability (MSI) status is recognized as a predictor of immunotherapy effectiveness, particularly in colorectal and gastric cancers [39]; however, its significance in bone metastases remains under investigation. The tumor immune microenvironment (TIME) plays a crucial role in influencing the success of immunotherapy. Studies indicate that bone metastases commonly have an immunosuppressive TIME characterized by regulatory T cells (Tregs), myeloid-derived suppressor cells (MDSCs), and tumor-associated macrophages (TAMs), which collectively inhibit anti-tumor immune responses [35]. A study focusing on bone metastases in NSCLC found that tumors with increased CD8^+^ infiltration and reduced MDSC levels were more likely to respond favorably to PD-1 inhibitors [75]. Additionally, genomic markers have been proposed as potential indicators. Greater sensitivity to PD-1 inhibition has been associated with lung cancer when KRAS and TP53 mutations coexist [75]. However, resistance to ICIs is associated with mutations that impair the function of PTEN and STK11 [73]. Incorporating these genomic markers into clinical decision-making could improve the selection of patients for immunotherapy involving bone metastases. Liquid biopsies have gained traction as non-invasive methods for monitoring responses to immunotherapy. Circulating tumor DNA (ctDNA) and circulating tumor cells (CTCs) provide timely insights into tumor evolution and the dynamics of the immune response [39]. Research has shown that a decline in ctDNA levels following ICI treatment correlates with extended survival in patients with metastatic lung and prostate cancer [39]. Furthermore, identifying immune-related gene signatures in peripheral blood could help predict the response to ICIs [39]. Despite their potential, liquid biopsy biomarkers face challenges, including inconsistent detection sensitivity and the need for standardization across various cancer types [39].

While biomarkers such as PD-L1 expression, tumor mutational burden (TMB), and microsatellite instability (MSI) have proven effective in predicting responses to immunotherapy, their effectiveness in bone metastases is complicated by the unique immunosuppressive properties of the bone microenvironment [39]. The diversity of bone metastases across various cancer types adds to the challenge of validating these biomarkers [74]. Furthermore, the interaction between bone-targeted treatments and immunotherapy remains an ongoing area of research. Agents such as bisphosphonates and RANKL inhibitors affect bone remodeling and immune interactions, which may impact the effectiveness of immune checkpoint inhibitors (ICIs) [40]. Understanding these interactions could assist in developing combination strategies that enhance treatment outcomes. The efficiency of predictive biomarkers for immunotherapy in bone metastases relies on incorporating multi-omic strategies. Integrating genomic, transcriptomic, and proteomic information with liquid biopsy and imaging markers could establish a comprehensive system for categorizing patients [39]. Moreover, advancements in machine learning algorithms for analyzing these datasets hold potential for improving predictive accuracy [73]. Future clinical trials assessing biomarker-guided immunotherapy approaches are crucial for validating and implementing these findings in clinical practice. As our understanding of the tumor–immune–bone axis advances, identifying robust predictive biomarkers will be essential for enhancing the efficacy of immunotherapy in patients with bone metastases [73].

#### 3.5.3. Predictive Genomic Signatures

Identifying predictive genomic markers has emerged as a hopeful approach for assessing the risk of bone metastasis (BM) across various cancer types. Genetic alterations influence tumor progression, metastatic potential, and responses to therapy. Utilizing genomic biomarkers to predict bone metastasis enables the early identification of high-risk patients, thereby encouraging timely treatment interventions [76]. Nonetheless, the clinical application of these biomarkers faces challenges, including variations in tumor heterogeneity, the reliability of the findings, and the availability of genomic testing.

Breast cancer is among the most common cancers linked to bone metastases, and many genomic markers have been recognized as possible indicators of bone dissemination. A significant example is DOCK4 (Dedicator of Cytokinesis 4), identified as a biomarker for assessing the risk of bone metastasis in early-stage breast cancer. Heightened DOCK4 expression is associated with increased invasiveness and a higher risk of metastatic recurrence in the bone. In a tissue microarray analysis from the AZURE trial, individuals with elevated DOCK4 levels in primary breast tumors showed markedly higher rates of bone recurrence than those with reduced DOCK4 expression. Interestingly, patients treated with zoledronic acid exhibited no such connection, implying that this bone-focused treatment might mitigate the heightened risk linked to DOCK4 overexpression. This suggests that DOCK4 could act as a biomarker for categorizing patients likely to benefit from bisphosphonate treatment, in addition to facilitating the early detection of high-risk individuals [77].

Moreover, a liquid biopsy panel targeting multiple genes has been developed to identify patients at elevated risk for bone metastases. Through the analysis of mRNA derived from exosomes, researchers have discovered a group of 15 genes that are consistently overexpressed in patients with metastatic breast and lung cancer. Among these, five genes demonstrated notable predictive accuracy in distinguishing between patients with and without bone metastases. The liquid biopsy technique provides a non-invasive approach for detecting genomic alterations that indicate bone metastasis, serving as a crucial tool for monitoring disease progression and informing treatment strategies. Nonetheless, the clinical use of this method remains limited due to the need for further verification in larger patient populations [78].

Markers of bone formation and resorption, such as alkaline phosphatase (ALP), osteopontin (OPN), and osteocalcin (OC), have demonstrated potential as genetic indicators of bone metastasis in prostate cancer. Increased ALP levels are associated with a greater tumor load in the skeletal system, and OPN is thought to play a role in the adhesion and persistence of metastatic cells within the bone microenvironment. However, while these markers provide valuable prognostic insights, their specificity presents a difficulty, as high ALP levels can arise from benign bone disorders or various cancers impacting bones [40].

Liquid biopsy techniques, particularly those analyzing circulating tumor DNA (ctDNA) and microRNA (miRNA) profiles, have been explored for predicting bone metastases in prostate cancer. For instance, distinct miRNA profiles have been discovered that can differentiate patients with localized prostate cancer from individuals who have bone metastases. Integrating these genomic biomarkers into clinical environments might enhance early detection; however, challenges remain in standardizing assay methods and ensuring consistency among different patient populations [40].

Lung cancer often spreads to bones, impacting about 30–40% of patients with advanced lung cancer who experience skeletal issues. Recent research has identified exosomal microRNAs (exo-miRNAs) as promising indicators for predicting bone metastasis in lung cancer. High-throughput sequencing has revealed multiple miRNAs that exhibit varying expression levels in lung cancer cell lines with notable metastatic potential in contrast to those without. Among the leading candidates, hsa-miR-151a-3p and hsa-miR-877-5p have demonstrated significant predictive ability in distinguishing between lung cancer patients with bone metastases and those without. These miRNAs play a role in cancer apoptosis and metabolic functions, highlighting their significance in the advancement of metastasis [79].

Although they hold potential, applying exosomal miRNAs as predictive biomarkers in clinical environments remains in its initial phases. Variations in methods for isolating exosomes and the influence of tumor heterogeneity create challenges for consistency. Moreover, although miRNA-based liquid biopsy tests provide non-invasive benefits, their sensitivity and specificity need additional validation in larger groups before they can be widely used in clinical practice [79].

Renal cell carcinoma (RCC) frequently metastasizes to the bone; nevertheless, reliable predictive biomarkers for this event remain scarce. A recent study identified a novel biomarker pattern associated with the likelihood of bone metastasis in patients with RCC. Utilizing a multiplex real-time RT-PCR assay, investigators assessed the expression of genes associated with angiogenesis and tumor invasion, discovering that VEGFR-1, VEGFR-2, HIF-1α, uPA, and PAI-1 were significantly upregulated in RCC patients with bone metastases when compared to those without. These findings suggest that tumor cells showing heightened angiogenic and invasive traits are more likely to invade the bone microenvironment. However, the application of these biomarkers in routine clinical environments is currently limited because they need validation in larger and more diverse patient populations [80].

Although genetic markers hold great potential for predicting bone metastases, several challenges hinder their widespread application in clinical settings. Tumor heterogeneity poses a significant challenge since different tumors, even within the same cancer type, have unique genetic characteristics. Technical constraints in genomic testing, such as sequencing depth, bioinformatics methods, and uniformity of assay protocols, can affect the reliability of results. Moreover, availability and cost remain concerns, particularly in settings with restricted resources where comprehensive genetic profiling may not be feasible.

Despite these challenges, ongoing advancements in liquid biopsy techniques, next-generation sequencing (NGS), and bioinformatics enhance the feasibility of integrating genomic biomarkers into clinical settings. Future research should focus on comprehensive validation studies, the incorporation of multi-omics techniques, and the standardization of genomic testing to improve these biomarkers’ predictive accuracy and clinical significance.

### 3.6. Monitoring Biomarkers for Treatment Efficacy in Bone Metastasis

Several circulating biomarkers have been recognized as possible indicators for evaluating treatment response, particularly circulating tumor cells (CTCs), circulating tumor DNA (ctDNA), and serum markers of bone resorption like tartrate-resistant acid phosphatase 5b (TRACP5b) and the C-terminal telopeptide of type I collagen (CTX). While these biomarkers provide vital insights into disease progression and treatment efficacy, their application in clinical practice encounters particular challenges.

#### 3.6.1. Circulating Tumor Cells (CTCs) and Circulating Tumor DNA (ctDNA)

CTCs and ctDNA serve as dynamic biomarkers for monitoring treatment efficacy across various cancers. A reduction in CTCs often signals a positive response to systemic therapies, while ctDNA enables real-time tracking of molecular responses and emerging resistance mutations, guiding personalized treatment adjustments [56].

Rizzo et al. highlighted the benefits of ctDNA analysis over traditional imaging techniques, emphasizing how it can identify changes in tumor load and minimum residual disease [56]. However, because bone metastases shed less tumor material into the bloodstream than soft tissue metastases, the sensitivity of ctDNA to bone metastases presents a challenge despite its potential as a non-invasive monitoring method [56]. Furthermore, while decreases in CTC count and ctDNA levels may indicate a favorable response to treatment, their persistence or recurrence may indicate treatment resistance or disease progression, requiring immediate clinical intervention [80].

#### 3.6.2. TRACP5b as a Biomarker for Treatment Monitoring

Feng et al. found that TRACP5b levels are significantly elevated in breast cancer patients who have bone metastases versus those who do not, and its expression is associated with tumor burden and the effectiveness of treatment [81]. Likewise, Chung et al. showed that TRACP5b activity serves as a more reliable indicator of treatment efficacy than traditional bone turnover markers such as bone alkaline phosphatase (BAP), particularly in patients undergoing bisphosphonate therapy [82]. Their findings revealed that TRACP5b levels significantly dropped in patients responding well to treatment, highlighting its significance as a crucial biomarker for assessing treatment outcomes [82].

Despite its benefits, TRACP5b also has limitations. Differences in assay standardization and potential alterations from non-tumor-related bone remodeling processes can affect its specificity as a monitoring tool. Additionally, relying solely on TRACP5b may not be sufficient to distinguish between disease progression and treatment-induced fluctuations in bone turnover, necessitating its use in conjunction with other biomarkers [81,82].

#### 3.6.3. C-Terminal Telopeptide of Type I Collagen (CTX) and Other Bone Resorption Markers

Despite its potential, CTX has challenges associated with its use as a biomarker. Although decreases in CTX levels typically suggest a positive response to anti-resorptive therapy, they do not always correspond with a significant reduction in tumor size, as changes in bone turnover can occur even when the tumor remains stable. This analysis is made more complex by differing baseline CTX levels in patients and variations in bone metabolism [83]. In clinical settings, circulating biomarkers like CTX, TRACP5b, ctDNA, and CTCs can quickly assess the effectiveness of treatments for bone metastases. Unlike traditional imaging methods, which often struggle to detect early responses due to the flare effect or limited sensitivity to micro-metastatic changes, these non-invasive biomarkers offer a considerable advantage [84]. Furthermore, utilizing various biomarkers could enhance diagnostic accuracy and provide a comprehensive evaluation of disease progression and treatment efficacy.

However, additional challenges remain to be tackled. Establishing standardization, validating outcomes through extensive clinical trials, and determining recognized cutoff values are essential to enhance the reliability and clinical application of biomarker tests. Additionally, different cancer types exhibit distinct patterns in bone metastases, necessitating tailored approaches for employing biomarkers to ensure precise patient categorization and individualized treatment strategies [35].

Circulating biomarkers such as CTCs, ctDNA, TRACP5b, and CTX provide valuable information on treatment effectiveness in bone metastasis, their clinical importance should be assessed within a broader context of disease monitoring. Expected progress in biomarker research and technology will probably enhance their efficacy in informing treatment choices and improving patient outcomes. To provide a comprehensive summary of the biomarkers discussed in this section, a detailed table highlighting various categories, clinical relevance, and limitations is presented below (Table 1).

This table provides an overview of various biomarkers associated with bone metastases, categorized into diagnostic, prognostic, predictive, and monitoring biomarkers. It highlights the specific biomarkers under each category, their clinical relevance, and limitations. The table emphasizes the importance of integrating these biomarkers into precision oncology for enhanced detection, prognosis, therapeutic prediction, and monitoring of bone metastases.

## 4. Advances in Precision Medicine for Bone Metastases

The advent of precision medicine, driven by biomarker discovery, genomic profiling, and personalized therapeutics, has revolutionized diagnostic and treatment strategies. This section explores recent advancements in genomic approaches, emerging therapeutics, and personalized treatment protocols for bone metastases (Table 2).

### 4.1. Genomic Approaches

#### 4.1.1. Next-Generation Sequencing (NGS) in Bone Metastases

Rapid sequencing of millions of DNA fragments at once is made possible by NGS (next-generation sequencing), which offers comprehensive knowledge into gene expression levels, genomic structure, genetic variants, and epigenetic changes [85]. Mutation-specific therapies have achieved remarkable success over the past two decades [86], driven by advancements in NGS, which has also enabled precision medicine strategies and enhanced diagnostic techniques [85].

Liquid biopsies enable individualized, dynamic therapy approaches, including diagnosis, prognosis, treatment selection, minimal residual disease detection, progression prediction, and resistance to various therapies, as well as cancer monitoring [87]. Compared to traditional tissue biopsies, liquid biopsies require smaller samples and allow for real-time tracking of tumor dynamics, facilitating personalized treatment adjustments. They analyze tumor-derived components in bodily fluids, including circulating tumor cells (CTCs), cell-free DNA (cfDNA), circulating tumor DNA (ctDNA), non-coding RNAs (ncRNAs), exosomes, and proteins [88].

#### 4.1.2. NGS in Clinical Trials and Treatment Selection

A comprehensive genomic profiling (CGP) report lists all actionable genetic changes in the patient and possible treatments [89]. CGP utilizing NGS may identify all classes of genomic abnormalities and is increasingly utilized to select patients for targeted therapy [90,91]. Molecular tumor boards (MTBs) identify actionable mutations by analyzing complete genomic profiling (CGP) results to incorporate NGS into clinical practice successfully [92]. NGS-based profiling is feasible and effective in clinical research, with recommendations for sequence-directed therapy in 63% of cases. The clinical significance of genomic profiling in advanced cancer therapy has been demonstrated by substantial survival advantages and a high disease control rate (65%) in patients undergoing MTB-guided treatment [92]. The effectiveness of a single targeted treatment is assessed in basket trials for various cancer forms with a common genetic mutation, independent of the tumor’s origin. One basket trial that employs NGS to find individuals with particular genetic mutations and match them with appropriate targeted medicines is the NCI-MATCH study (National Cancer Institute Molecular Analysis for Therapy Choice) [93]. On the other hand, umbrella trials evaluate several targeted medicines based on distinct genetic changes within a single cancer type. In the ALCHEMIST trial, for instance, patients with non-small-cell lung cancer (NSCLC) undergo NGS testing for certain genetic alterations, and treatment choices are based on the results [93].

#### 4.1.3. Multi-Omics Integration (Proteomics and Metabolomics)

Proteomics is a methodical research strategy widely used in pathogenetic studies, including tumor metastasis and biomarker development. Its goal is to evaluate protein expression and function globally under certain conditions [94]. This method enables the identification of protein biomarkers that differentiate between osteolytic (bone-resorbing) and osteoblastic (bone-forming) metastases, which is essential for determining the prognosis and course of treatment [41]. The bone microenvironment significantly influences the progression of bone metastases [95]. It has been shown that proteins such as osteopontin (OPN) mediate important interactions between tumor cells and the bone microenvironment, facilitating the colonization and proliferation of metastatic cells inside the bone. Critical phases in the metastatic cascade, such as cell adhesion, migration, invasion, and bone remodeling, are influenced by OPN. Knowing their function can help identify new therapeutic targets to break the metastatic niche and stop bone metastases from spreading [96]. One major obstacle to treating bone metastases is drug resistance [97]. One study that used mass spectrometry-based proteomics found that serpin B9 (SB9) plays a crucial role in bone metastasis. In individuals with lung cancer, overexpression of SB9 was linked to a worse prognosis and a higher burden of bone metastases. This implies that SB9 might mediate resistance to treatments that target bone metastases [98]. Proteomic studies have also shown that drug-resistant cancer cells can interact with sensitive cells to impart resistance characteristics. Changes in protein expression profiles, including the elevation of proteins linked to drug resistance pathways, are part of this phenomenon. Knowing these relationships helps one better understand how resistance might spread among tumor populations, possibly resulting in treatment failure [99]. The development of bone metastases is significantly influenced by metabolic reprogramming, especially in the hypoxic bone marrow microenvironment. When oxygen is scarce, this metabolic change promotes tumor growth and survival [100]. To meet rapid growth needs, cancer cells often exhibit enhanced glutamine absorption. As a flexible substrate, glutamine aids in the production of amino acids and nucleotides and the tricarboxylic acid cycle (TCA cycle) [101]. Changes in lipid metabolism, such as increased oxidation of lipids, also provide energy and building blocks for membrane production, promoting metastasis formation [102].

The acidic nature of the tumor microenvironment is one of its distinguishing characteristics, and it is essential for cellular interactions and signaling during carcinogenesis. Lactate is the primary contributor of this acidity as cancer cells constantly release protons and lactate into the extracellular space, resulting in an acidic microenvironment [103]. Potential therapeutic targets have been identified through an understanding of these metabolic processes. For instance, it has been demonstrated that blocking glutaminase, the enzyme that converts glutamine, can interfere with tumor metabolism [104]. Therapeutic intervention options include targeting the pathways involved in lipid metabolism and lactate generation, aiming to disrupt the metabolic flexibility cancer cells utilize to survive and proliferate.

### 4.2. Emerging Therapeutics

#### 4.2.1. Targeted Therapies: Precision Treatment in Bone Metastases

By targeting specific molecular pathways implicated in tumor growth and bone resorption, targeted treatments have made substantial progress in the management of bone metastases. The protein RANKL, necessary for osteoclast activation, is targeted and neutralized by the completely human monoclonal antibody denosumab. In patients with bone metastases, denosumab effectively decreases osteoclast-mediated bone resorption by blocking the RANKL-RANK interaction, thereby lowering skeletal-related events (SREs) [105]. Clinical trials have demonstrated that denosumab is superior to the commonly administered bisphosphonate zoledronic acid in delaying the first on-study skeletal-related event (SRE) and reducing the risk of subsequent SREs in patients with advanced cancers that involve bone metastases [106]. Current clinical practice rarely evaluates RANKL expression levels as a predictive biomarker for therapy response, despite denosumab directly targeting the RANKL pathway. Ongoing research aims to identify biomarkers predicting optimal responders to RANKL inhibition [107].

#### 4.2.2. Tyrosine Kinase Inhibitors (TKIs) in Bone Metastases

Tyrosine kinase inhibitors (TKIs), which target key signaling pathways involved in tumor growth and bone metastasis, have become attractive treatment options. Alterations in the fibroblast growth factor receptor (FGFR) are linked to a number of cancers, contributing to the growth and spread of malignancies. In patients with advanced solid tumors with FGFR mutations or fusions, erefitinib, an oral pan-FGFR TKI, has shown success. The RAGNAR phase 2 research showed promise in treating FGFR-mutant malignancies, including those with bone metastases, with an objective response rate (ORR) of 30% across 16 different tumor types [108]. 

Tumor development, survival, and the interaction between tumor cells and the bone microenvironment are significantly influenced by the PI3K/AKT/mTOR pathway. By interfering with bone–tumor crosstalk, inhibitors that target this system may be able to stop the spread of bone metastases [109]. Bisphosphonates, anti-resorptive drugs, are frequently used to treat bone metastases in multiple myeloma, prostate cancer, and breast cancer. They lessen skeletal-related events and bone pain by preventing osteoclast-mediated bone resorption [110]. The results of patients with ALK-positive non-small-cell lung cancer (NSCLC), including those with bone metastases, have been markedly enhanced by anaplastic lymphoma kinase (ALK) inhibitors. Alectinib, a second-generation ALK inhibitor, is more effective and less toxic than crizotinib when used as the primary treatment for ALK-positive non-small-cell lung cancer. According to the ALEX study, alectinib considerably increased progression-free survival (PFS) compared to crizotinib, with a median PFS of 34.8 months compared to 10.9 months [111]. According to research, bone metastases are prevalent in individuals with ALK-positive non-small-cell lung cancer (NSCLC), accounting for roughly 43% of cases [112]. Although there are not many trials specifically addressing bone metastases, the effectiveness of ALK inhibitors like alectinib in managing systemic disease has been demonstrated.

#### 4.2.3. Bisphosphonates in Bone Metastases

Anti-resorptive drugs called bisphosphonates, like zoledronic acid and pamidronate, are frequently used to treat bone metastases in a variety of malignancies, such as multiple myeloma, breast, and prostate cancer. They work by preventing osteoclasts from resorbing bone, which lessens skeletal-related incidents and eases bone pain [113].

#### 4.2.4. Emerging Nanoformulations for Enhanced Bone Targeting

Nanoformulations have been developed through advancements in nanotechnology to enhance the effectiveness and distribution of bisphosphonates. One study, for example, created a formulation of nanoparticles containing zoledronic acid that reduced premature drug release and improved efficacy against extraskeletal malignancies. These nanoparticles showed decreased transport to bones and enhanced cytotoxicity against tumor cells, suggesting that they may be used to treat extraskeletal and bone metastases [114]. An additional novel strategy for targeted bone distribution involved linking zoledronic acid to mesoporous silica nanoparticles. This drug delivery method showed promise for treating bone metastases in vitro with its pH-sensitive drug release, efficient cellular uptake, and notable suppression of tumor cell migration [115].

These developments highlight the potential benefits for patients with bone metastases from ALK-positive NSCLC and other malignancies who may benefit from combining innovative bisphosphonate nanoformulations with next-generation ALK inhibitors.

#### 4.2.5. Radiopharmaceuticals: Bone-Targeted Therapy

An alpha-emitting radiopharmaceutical called radium-223 dichloride is authorized for the treatment of castration-resistant prostate cancer (CRPC) that has bone metastases. Radium-223 delivers high-energy alpha particles that cause double-stranded DNA breaks in cancer cells, leading to cell death, and selectively accumulates in regions of accelerated bone turnover, such as bone metastases, due to its chemical resemblance to calcium. According to clinical trials, radium-223 dramatically increases overall survival and postpones skeletal-related outcomes in patients with CRPC who have bone metastases [116]. Lutetium-177 (Lu-PSMA-617) is a beta-emitting radionuclide therapy that targets bone metastases. Beta radiation can be delivered straight to tumor cells expressing PSMA using this focused strategy. According to recent research, Lu-PSMA-617 therapy significantly lowers prostate-specific antigen (PSA) levels and offers clinical advantages to individuals with metastatic CRPC, particularly those whose bones are affected [117]. The combination of radiopharmaceuticals with other treatment modalities, such as immunotherapy or chemotherapy, is being explored to enhance therapeutic efficacy. Patients with metastatic castration-resistant prostate cancer may benefit better from pembrolizumab in conjunction with ^177^Lu-PSMA-617 than from conventional therapy [118]. The efficacy and safety of these combined techniques are being evaluated in ongoing clinical trials.

#### 4.2.6. Immunotherapeutics in Bone Metastases

Depending on the metastatic site, immune checkpoint inhibitors (ICIs) have demonstrated different response rates. Research shows that compared to patients with visceral metastases, those with bone metastases have worse overall response rates (ORRs) to ICIs [119]. For example, one study found that individuals with bone metastases had an ORR of 16%, while those without bone involvement had an ORR of 41% [120]. The immunosuppressive properties of the bone marrow microenvironment present special difficulties for immunotherapy. The expression of RANKL and TNFα by non-activated T lymphocytes invading bone metastases can increase osteoclastogenesis and stimulate osteolytic activity. On the other hand, activated T cells prevent the development of osteoclasts by producing IL-4 and IFNγ. Consequently, methods that stimulate T lymphocytes in the bone microenvironment may be able to fight cancer cells and slow down bone resorption [121]. B-cell maturation antigen (BCMA) is the target of chimeric antigen receptor (CAR) T-cell therapy, which has become a game-changing treatment for multiple myeloma, a cancer often associated with bone disease. According to clinical research, individuals with relapsed or refractory multiple myeloma can have notable clinical improvements when BCMA-targeted CAR-T cells successfully destroy myeloma cells [122]. Bispecific T-cell engagers (BiTEs) are synthetic compounds that bind simultaneously to both tumor and T cells, directing the T cells to the tumor cells. Recent developments have produced BiTEs, which improve the immune system’s capacity to identify and eradicate cancer cells found in bone tissue by targeting particular antigens expressed in bone metastatic niches [123]. 

### 4.3. Personalized Treatment Protocols

#### 4.3.1. Case Studies and Clinical Trials

Numerous clinical trials have investigated the use of precision medicine in treating bone metastases. For instance, in patients with metastatic castration-resistant prostate cancer (mCRPC) with homologous recombination repair gene alterations, including those with bone metastases, olaparib dramatically increased radiographic progression-free survival and overall survival, according to the PROfound trial [124]. The TAPUR study is a non-randomized clinical trial that assesses the effectiveness of targeted medicines licensed by the FDA in patients with advanced malignancies with genomic abnormalities. The trial’s design enables the evaluation of targeted treatments across different tumor types and metastatic sites, despite the lack of particular data on bone metastases [125]. In the precision medicine trial Molecular Analysis for Therapy Choice, or NCI-MATCH, cancers are treated according to their genetic abnormalities rather than where they are located in the body. By including patients with bone metastases, the efficacy of targeted therapy in this particular cohort can be investigated [126]. Regarding BRCA-mutated prostate cancer with bone metastases in patients with mCRPC who had homologous recombination repair gene changes, the phase 3 PROfound trial compared olaparib to enzalutamide or abiraterone. The study demonstrated that olaparib significantly increased overall survival, including in individuals with bone metastases, as well as radiographic progression-free survival [127]. The effectiveness of targeted medicines in diverse patient populations must be evaluated using adaptive trial designs incorporating predictive biomarkers. By identifying subgroups that may benefit from specific therapies, such designs can enhance the clinical utility of trials [128].

#### 4.3.2. Incorporation of Biomarkers into Therapeutic Algorithms

The National Comprehensive Cancer Network (NCCN) and the European Society for Medical Oncology (ESMO) have integrated molecular profiling into their guidelines for managing bone metastases [129]. These guidelines emphasize using specific biomarkers to guide therapy decisions, enhancing personalized treatment approaches [129]. Proteomics, radiomic AI models, and next-generation sequencing (NGS) are essential for risk stratification and treatment selection for patients with bone metastases. For example, by extracting quantitative information from medical images, radiomics helps anticipate how a disease will advance and how well a treatment will work. A more accurate and customized approach to patient treatment is made possible by the integration of these technologies [130].

#### 4.3.3. Future Directions in AI-Driven Precision Medicine

Machine learning algorithms are being developed to predict how patients with bone metastases will respond to specific therapies. These algorithms can optimize therapy tactics by identifying patterns and biomarkers that indicate favorable or negative reactions to therapies by evaluating massive datasets [130]. AI-driven liquid biopsy shows promise for non-invasive monitoring of bone metastases, offering insights into disease progression and treatment response. However, challenges persist in biomarker detection and isolation. Combining AI with liquid biopsy could enhance its accuracy, but it still needs to be complemented with tissue biopsy for a full understanding of bone metastases [131,132]. These developments herald a shift toward AI-driven precision medicine, in which genetic profiling and machine learning algorithms combine to tailor therapies for patients with bone metastases, ultimately enhancing treatment outcomes.

### 4.4. Clinical Challenges of Managing Bone Metastases

Clinical management of bone metastases poses several challenges affecting patient quality of life (QoL) and healthcare costs. One of the primary clinical challenges is the high incidence of skeletal-related events (SREs), such as pathologic fractures, spinal cord compression, and the necessity for palliative radiotherapy or surgical interventions. These events not only contribute to significant pain and reduced mobility but also lead to a decline in the overall QoL for patients, frequently necessitating aggressive symptom management strategies [133].

Although bone-targeted therapies, such as intravenous bisphosphonates and denosumab, have been developed to reduce the incidence of SREs, their efficacy is sometimes limited by the advanced tumor burden and the multifocal nature of bone metastases. Surgical interventions, while valuable for single or localized lesions, often remain impractical in patients with multiple metastatic sites [134]. The heterogeneity in clinical presentation among patients, with some facing significant bone pain, nerve compression syndromes, or structural instability, requires a highly individualized, multidisciplinary treatment approach. This variety in clinical course also complicates decision-making regarding the timing and type of intervention, thus affecting both short-term and long-term patient management [135,136].

Managing bone metastases significantly increases healthcare resource utilization and associated costs. The frequent need for diagnostic imaging such as bone scintigraphy and PET/CT scans, regular outpatient visits, and interventional procedures all contribute to an elevated economic burden within healthcare systems [137]. In several studies, outpatient visits and radiotherapy sessions have been identified as major contributors to the incremental costs incurred by patients with bone metastases compared to those without such complications [137]. This increased financial strain not only affects the sustainability of health services but also poses challenges in ensuring equitable access to the most effective therapeutic modalities, particularly in resource-constrained settings [138].

## 5. Challenges and Limitations

### 5.1. Heterogeneity of Bone Metastases

The variation in bone metastases presents a major hurdle in establishing and utilizing pathological biomarkers for precision medicine. Bone metastases stem from a range of primary tumors, including breast, prostate, and lung cancers, each displaying specific biological and molecular features [139,140]. 

This diversity is reflected in the bone microenvironment, where differing osteoclast and osteoblast activities, bone remodeling processes, and local factors such as cytokines and growth factors play a role in tumor progression variability [141]. The complexity of these interactions undermines the reliability and reproducibility of pathological biomarker assays, complicating the development of standardized diagnostic tests and predictive biomarkers [142].

The variability in molecular pathways, including RANK/RANKL/OPG, TGF-β, and IGF signaling, differs among metastatic lesions, hindering the development of universally applicable biomarkers [141]. Moreover, the presentations of metastatic lesions—osteoblastic, osteolytic, and mixed phenotypes—pose technical difficulties in effectively capturing the entire spectrum with imaging and molecular techniques [139]. 

This heterogeneity results in inconsistent treatment responses, complicating the customization of precision medicine for individual patients [143]. Differences in biomarker expression among various metastatic sites, as well as changes within the same patient over time, require comprehensive multi-omics and dynamic monitoring strategies [142].

To address these challenges, developing comprehensive diagnostic platforms that account for these variations and refining personalized treatment protocols are crucial. These platforms should integrate various biomarkers, sophisticated imaging techniques, and ongoing monitoring to reflect the intricate and dynamic characteristics of bone metastases [45,144].

### 5.2. Standardization of Biomarker Assays

Standardizing biomarker assays presents significant technical and clinical challenges that hinder their integration into precision medicine for bone metastases. Variability in preanalytical and analytical processes is a key concern. Differences in specimen collection, handling, storage, and processing, such as fixation techniques or delays in preparation, can result in significant discrepancies in assay outcomes. These preanalytical factors adversely affect the sensitivity and specificity of biomarker measurements, reducing their reliability across various studies and clinical applications [145,146]. 

Furthermore, the influence of operator expertise and platform variability amplifies these challenges. Assays such as immunohistochemistry (IHC), fluorescent in situ hybridization (FISH), and molecular tests are particularly susceptible to inconsistencies arising from operator skill, laboratory protocols, and instrument calibration. The absence of standardized operating procedures across laboratories further intensifies these issues, making reproducibility a continuing concern [147,148].

Variations in defining positive cutoffs for biomarker assays and inconsistent scoring systems obstruct the comparability and validation of results across studies. These reproducibility issues complicate the clinical interpretation of biomarker data and slow the advancement of reliable predictive assays [148,149]. 

These challenges notably hinder clinical implementation because the absence of standardized assay protocols restricts the smooth incorporation of biomarker testing into everyday practice. This shortcoming impacts patient selection for targeted therapies and compromises personalized treatment decisions, ultimately diminishing the effectiveness of precision medicine in managing bone metastases [146,150].

### 5.3. Challenges of Translating Research Findings into Clinical Practice

Current biomarker-driven approaches for diagnosing and treating bone metastases have several intrinsic limitations that hinder their clinical utility. One major challenge relates to the specificity and sensitivity of biochemical and molecular markers. Traditional biochemical markers of bone metabolism, such as alkaline phosphatase or collagen peptide fragments, although indicative of altered bone remodeling, suffer from poor specificity and are influenced by multiple confounding factors, including non-malignant bone diseases and variability due to renal impairment [41]. Similarly, prostate-specific antigen (PSA) and other protein-based markers provide limited information regarding metastatic involvement and bone remodeling, thus failing to capture the heterogeneity of metastatic lesions [59].

Several technical and biological variables compromise the ease of translation from laboratory discovery to clinical validation. The inherent variability of candidate biomarkers like circulating tumor cells (CTCs) and microRNAs, compounded by issues with preanalytical sample processing and inter-assay variability, undermines reproducibility and diagnostic accuracy [151,152]. The complexity of the bone microenvironment, characterized by dynamic interplay among osteoblasts, osteoclasts, and various cytokines, adds another layer of biological variability that is not easily standardized in biomarker assays [153]. These factors result in difficulties when attempting to validate candidate biomarkers across distinct patient populations and cancer types, thereby slowing the incorporation of novel markers into routine clinical protocols.

### 5.4. Regulatory and Ethical Considerations

#### 5.4.1. Validation of Biomarkers for Clinical Use

The process of validating biomarkers for clinical use is intricate and requires careful analytical and clinical validation to ensure they are both reliable and useful. Analytical validation assesses the accuracy, precision, sensitivity, and specificity of biomarker assays. In contrast, clinical validation focuses on proving that these biomarkers can genuinely improve patient outcomes through well-structured clinical trials [154,155]. Creating standardized protocols, cutoff thresholds, and quality control measures that meet regulatory guidelines from the FDA or EMA is challenging. These standards are vital for achieving reproducibility in laboratories and ensuring consistent assay performance. Unfortunately, differing methodologies can complicate the regulatory approval process [155].

Regulatory challenges highlight the necessity for multi-center studies to assess biomarkers across various conditions and confirm their reliability [156,157]. Variations in assay performance or the absence of standardized methods can delay the approval process or lead to rejection. This emphasizes the need for consistent protocols and robust quality assurance practices [158]. Insufficient validation raises ethical issues, such as the risks of misdiagnosis, harm to patients, and financial burdens from ineffective treatments [159]. To reduce these risks and preserve trust in clinical practices, transparency in biomarker development and the process of obtaining informed consent are crucial [159,160].

Researchers, clinicians, regulatory bodies, and ethicists must collaborate across disciplines to tackle these challenges. This teamwork is essential for creating reliable, standardized, and ethically sound biomarker assays that can effectively guide precision medicine strategies [161,162].

#### 5.4.2. Ethical Implications of Genetic Testing

The ethical considerations surrounding genetic testing in precision medicine for bone metastases are quite complex. They include privacy concerns, informed consent, psychological effects, and the need for regulatory safeguards. Privacy and data protection are significant issues because genetic information is highly sensitive and at risk of unauthorized access. Data breaches can lead to discrimination from employers and insurance companies, despite laws such as the Genetic Information Nondiscrimination Act (GINA) being in place. GINA protects against discrimination in health insurance and employment, but it does not apply to life or long-term care insurance, leaving some gaps in coverage [163,164]. To mitigate these risks, implementing robust data security practices and having clear policies are essential.

Informed consent and patient autonomy are major challenges in helping patients understand their genetic test results. Patients must recognize the possible risks of developing future health conditions and the effects of sharing their genetic information. Comprehensive counseling is necessary to clarify uncertainties and support independent decision-making [165,166]. Informed consent frameworks need to balance the importance of actionable clinical insights with respect for patient autonomy, especially when incidental findings or family implications come into play [166].

The psychological and social effects of genetic testing can be considerable. Patients may feel anxiety, stress, or a shift in how they view themselves after learning about their genetic predispositions. This can result in stigma or tension in family relationships, particularly with hereditary conditions [167,168]. Furthermore, awareness of genetic risks may lead to feelings of fatalism or hopelessness, highlighting the importance of psychological support and clear communication regarding the limitations of predictive testing [168].

Finally, Ethical use and regulation require clear rules to ensure that genetic data serve clinical purposes only. It is essential to follow laws like GINA to prevent discrimination and foster trust in genetic testing [163,164]. Furthermore, ethical oversight should ensure fair access to testing and open discussions about its benefits and potential risks. Teamwork among healthcare providers, ethicists, and policymakers is key to addressing these issues while responsibly advancing precision medicine ethically [166,169].

## 6. Conclusions

Bone metastases represent a formidable challenge in cancer management, demanding multifaceted approaches for improved detection, prognosis, and treatment.

Liquid and tissue biomarkers, bone turnover assays, advanced imaging techniques, and tumor genomic profiling are now coming together to offer clinicians a unified and detailed picture of skeletal disease. These tools help clarify when metastases emerge, predict their aggressiveness, and identify optimal therapeutic targets. Leveraging this integrated approach can shorten diagnostic timelines, enhance risk assessment, and support more personalized treatment strategies. Achieving this potential, however, depends on standardized testing protocols, consistent data frameworks, and large-scale, multi-institutional studies that embed biomarkers into routine clinical oncology.

## Figures and Tables

**Table 1:** Summary of biomarkers in bone metastases.

Biomarker	Category	Cancer Type(s)	Clinical Relevance	Limitations
CTCs	Diagnostic, Prognostic, Monitoring	Breast, Prostate, Lung, NETs	Associated with metastases and survival; aids in diagnosis and treatment monitoring	Detection challenges due to heterogeneity and low abundance; EMT subtypes may escape detection
ctDNA	Diagnostic, Prognostic, Monitoring	Breast, Prostate, Lung	Non-invasive tumor profiling; tracks mutations and resistance; correlates with prognosis	Low levels in bone-dominant disease; false negatives; lack of standard thresholds
BALP	Diagnostic	Prostate	Marker of osteoblastic activity; diagnostic performance comparable to imaging	May be influenced by non-malignant bone conditions
P1NP	Diagnostic	Breast, Prostate	Indicates osteoblastic activity in metastatic lesions	Specificity limited due to elevations in other bone conditions
P1CP	Prognostic	Prostate	Linked to metastatic progression and survival	Requires further validation
Osteocalcin	Diagnostic	General	Reflects bone turnover	Affected by systemic conditions; low specificity
CTX	Diagnostic, Monitoring	Breast, Prostate	Assesses osteoclastic activity; reduced post-treatment	Does not always correlate with tumor burden
NTX	Diagnostic, Prognostic	Breast, Prostate	Correlates with disease severity and skeletal events	Affected by physiological bone turnover
DPD	Diagnostic	Breast, Prostate, Lung	Urinary marker of bone resorption	Variable results due to urinary excretion differences
TRACP-5b	Diagnostic, Monitoring	Breast, Prostate	Reliable resorption marker; unaffected by renal function	Assay standardization and specificity remain challenges
Imaging biomarkers (PET/CT, MRI, etc.)	Diagnostic	Prostate, Breast, Lung	Enhance metastasis detection and staging	Cost, accessibility, and modality-specific limitations
Bone matrix proteins (OPN, BSP)	Prognostic	Prostate, Lung	Elevated levels correlate with poor prognosis	Influenced by non-malignant conditions; needs validation
CTGF	Prognostic	HCC	Associated with increased risk of bone metastasis	Not validated across cancer types
IL-11	Prognostic	HCC	Linked to osteoclastogenesis and worse outcomes	Cancer-type-specific relevance
MMP-1	Prognostic	HCC	Marker for bone metastasis risk	Requires further clinical validation
lncRNAs (MALAT1, HOTAIR, ANCR, ZEB1-AS1)	Prognostic	Breast, Prostate, NSCLC	Linked to EMT, metastasis, and prognosis	Detection and sample stability are challenging
Bisphosphonate response	Predictive	Breast, Prostate	Response stratified by MAF status and menopausal state	Varying response across cancers; more markers needed
RANKL inhibitors (Denosumab)	Predictive	Prostate	Delays SREs; useful in treatment response prediction	No clear OS benefit; limited specificity
PD-L1	Predictive	NSCLC, Kidney, Melanoma	Correlates with ICI treatment response	Immunosuppressive bone environment reduces utility
TMB	Predictive	NSCLC	High TMB = better ICI response	Lower TMB in bone mets limits predictive value
MSI	Predictive	Colorectal, Gastric	Predicts ICI response	Limited evidence in bone mets
KRAS/TP53 (lung)	Predictive	NSCLC	Co-mutations linked to improved immunotherapy outcomes	Requires broader validation
PTEN/STK11 loss	Predictive	NSCLC	Associated with resistance to ICIs	Negative predictive marker; not universal
DOCK4	Predictive	Breast	Identifies patients who benefit from bisphosphonates	Effect may be negated by zoledronic acid
Exosomal miRNAs (miR-151a-3p, miR-877-5p)	Predictive	Lung	Differentiate patients with and without bone mets	Isolation and reproducibility challenges
VEGFR-1, VEGFR-2, HIF-1Î±, uPA, PAI-1	Predictive	RCC	Upregulated in bone mets; indicate aggressive behavior	Early-phase research; needs broader trials
aBSI (Automated Bone Scan Index)	Prognostic	Prostate	Quantitative scan-based biomarker; predicts survival	Expensive; requires standardization

**Table 2:** Clinically actionable biomarkers for bone metastases—matched therapies and strength of supporting evidence.

Biomarker (Sample/Modality)	Dominant Pathway/Target	Matched Drug or Clinical Decision	Highest Level of Evidence	Primary Utility
RANKL: OPG ratio (serum/tissue)	RANK–RANKL–OPG osteoclast axis	Start or intensify denosumab to delay skeletal-related events	Phase III; guideline endorsed for breast and prostate SRE prevention	Predictive/ Monitoring
C-telopeptide (CTX) (serum)	Bone resorption	Gauge response to bisphosphonates (zoledronic acid); falling CTX = biochemical response	Prospective biomarker–response correlations in metastatic models	Monitoring
TRACP-5b (serum)	Osteoclast activity	Early signal of bisphosphonate efficacy or biochemical progression	Multi-institutional real-world data (prostate) and breast cohorts	Monitoring
PD-L1 TPS/CPS (tissue)	PD-1/PD-L1 checkpoint	Select pembrolizumab, nivolumab, atezolizumab, etc.	Multiple Phase III ICI trials; FDA-approved companion assays in NSCLC and RCC	Predictive
ctDNA EGFR/KRAS/ALK (plasma)	Driver mutations and resistance mechanisms	Choose or switch EGFR/ALK TKIs (e.g., osimertinib) or clinical-trial enrollment	≥Phase II concordance/utility studies in metastatic NSCLC	Predictive/ Monitoring
DOCK4 over-expression (tumor)	Cytoskeletal GEF; metastasis biology	Identifies early-stage breast patients most likely to benefit from adjuvant bisphosphonate chemoprevention	AZURE tissue-microarray correlative study (exploratory)	Predictive
Automated Bone-Scan Index (aBSI) (^99m^Tc-MDP planar scan)	Quantitative tumor burden on bone scintigraphy	Prognostic stratification; informs timing of radium-223 or systemic intensification	Phase III secondary-analysis validation (mCRPC)	Prognostic
Serum VEGF (±uNTx)	Angiogenesis/hypoxia	Consider bevacizumab (±TKI) in high-VEGF, bone-dominant settings; monitor anti-angiogenic response	Pre-clinical bone-metastasis models; ongoing clinical trials	Monitoring/ Exploratory
